# Neural Progenitor Cells and the Hypothalamus

**DOI:** 10.3390/cells12141822

**Published:** 2023-07-11

**Authors:** Evanthia A. Makrygianni, George P. Chrousos

**Affiliations:** University Research Institute of Maternal and Child Health & Precision Medicine, National and Kapodistrian University of Athens, 11527 Athens, Greece

**Keywords:** neurogenic niche, neural progenitor cells, tanycytes, hypothalamic nuclei, circumventricular organs, neuropeptides, neurotransmitters, neural circuits, extracellular vesicles

## Abstract

Neural progenitor cells (NPCs) are multipotent neural stem cells (NSCs) capable of self-renewing and differentiating into neurons, astrocytes and oligodendrocytes. In the postnatal/adult brain, NPCs are primarily located in the subventricular zone (SVZ) of the lateral ventricles (LVs) and subgranular zone (SGZ) of the hippocampal dentate gyrus (DG). There is evidence that NPCs are also present in the postnatal/adult hypothalamus, a highly conserved brain region involved in the regulation of core homeostatic processes, such as feeding, metabolism, reproduction, neuroendocrine integration and autonomic output. In the rodent postnatal/adult hypothalamus, NPCs mainly comprise different subtypes of tanycytes lining the wall of the 3^rd^ ventricle. In the postnatal/adult human hypothalamus, the neurogenic niche is constituted by tanycytes at the floor of the 3^rd^ ventricle, ependymal cells and ribbon cells (showing a gap-and-ribbon organization similar to that in the SVZ), as well as suprachiasmatic cells. We speculate that in the postnatal/adult human hypothalamus, neurogenesis occurs in a highly complex, exquisitely sophisticated neurogenic niche consisting of at least four subniches; this structure has a key role in the regulation of extrahypothalamic neurogenesis, and hypothalamic and extrahypothalamic neural circuits, partly through the release of neurotransmitters, neuropeptides, extracellular vesicles (EVs) and non-coding RNAs (ncRNAs).

## 1. Introduction

Stem cells may be undifferentiated (pluripotent) or partially differentiated (multipotent) [1]. Pluripotent stem cells have the potential to differentiate into all cell types of an organism, whereas multipotent stem cells, also termed “progenitor cells”, can give rise to specific subsets of cell types [1]. Embryonic stem cells (ESCs) (cells derived from the inner cell mass of the blastocyst) are pluripotent, whereas the embryonic layers of a specific tissue arise and develop from cell divisions of progenitor cells [2]. Stem cell divisions can be symmetric or asymmetric [3]. In the former, a stem cell divides into two identical cells that are destined to acquire the same cell fate [3]. In the latter, a stem cell divides into one daughter cell with a stem-cell fate and another one that is more differentiated [3]. The purpose of symmetric divisions is self-renewal and proliferation, whereas that of asymmetric divisions is the maintenance of cell number and differentiation [3]. The majority of stem cells can alternate between symmetric and asymmetric divisions [3]. The balance between the symmetric and asymmetric modes of division is regulated by intrinsic and extrinsic signals [3]. Perturbations in this balance may lead to tissue and organismal dyshomeostasis and/or disease [3].

Neural progenitor cells (NPCs) are multipotent neural stem cells (NSCs) that can self-renew and differentiate into neurons and glial cells [4] except for microglial cells, which are immune cells of mesodermal/mesenchymal origin that migrate into the CNS [5]. NPCs are present in the embryonic central (CNS) and peripheral nervous system (PNS) [1,4]. Of note, portions of the cranial nerves originate from neural crest cells (NCCs), which are transient ESCs of the developing dorsal neural tube that undergo rapid induction and specification, characterized by changes in gene expression and protein localization [6,7]. These modifications allow NCCs to separate from neighboring epithelial cells and migrate to distant sites of the developing embryo, giving rise to NCC derivatives, including Schwann cells, sympathetic ganglia and dorsal root sensory ganglia [6,7]. This process is known as epithelial-mesenchymal transition (EMT) [6,7].

NPCs can be in vitro generated from ESCs or induced pluripotent stem cells (iPSCs) [1]. iPSCs can be generated from adult cells (usually fibroblasts or blood cells) after their reprogramming into pluripotent stem cells that resemble ESCs [1].

NPCs have been identified in the postnatal and adult brain [1,4]. The characterization of adult vs. embryonic NPCs is based on various characteristics including spatiotemporal distribution, structure, function and expression of specific molecular markers [1]. NPCs in the embryonic brain have a greater potential for differentiation than NPCs in the adult brain [1]. In the postnatal/adult brain, NPCs are mainly located in the subventricular zone (SVZ) of the lateral ventricles (LVs) and the subgranular zone (SGZ) of the hippocampal dentate gyrus (DG), which constitute the “traditional adult neurogenic niches” [8]. In rodents, SVZ NPCs give rise to immature neurons that migrate along the rostral migratory stream (RMS) and reach the olfactory bulb (OB), where they differentiate into mature neurons that process olfactory input signals [9]. On the other hand, SGZ NPCs generate granule cells that process information associated with memory and learning [10].

A neurogenic niche is the highly complex local physiological milieu that supports and modulates the developmental trajectory, maintenance and differentiation of NPCs in the nervous system [11]. This structure consists of NPCs and their progeny, glial cells (including ependymal cells), pericytes and vascular cells [11,12]. Astrocytes play a crucial role in various aspects of neurogenesis, including cellular self-renewal, fate specification, differentiation, migration, and synaptic integration [11,12]. In the postnatal/adult SVZ, ependymal cells modulate the quiescent state and self-renewal of NPCs, whereas, in the SGZ, neurogenesis is spatiotemporally regulated by the activity of neighboring neurons that act as niche cells [12]. Vascular cells contribute to the regulation of the proliferation of adult NPCs by modulating the permeability of the contact between NPCs and vascular cells [12]. During embryogenesis, pericytes are mainly involved in the maturation of the blood-brain barrier (BBB) [13] by influencing angiogenesis (through the secretion of transforming growth factor beta (TGFβ) and insulin-like growth factor 2 (IGF2)) and neurogenesis (by limiting the access of blood-borne signaling molecules to the NPCs) [11,14,15]. It remains unknown whether pericytes also exert a direct effect on neurogenesis [11].

There is evidence that postnatal/adult neurogenesis takes place in other CNS regions besides the SVZ and SGZ, such as the neocortex, amygdala, striatum, cerebellum, spinal cord and hypothalamus [1,4,8,12,16]. In the periventricular zone of the spinal cord (PVZ), which is the spinal cord developmental analog of the brain SVZ, the neurogenic niche consists of NPCs, multiciliated ependymal cells, cerebrospinal fluid (CSF)-contacting cells, and tanycytes; and is influenced by astrocytes, oligodendrocyte progenitor cells (OPCs), microglia, macrophages, and matrix components [17]. Following spinal cord injury, the PVZ niche microenvironment undergoes modifications that collectively influence the NPC pool [17]; PVZ NPCs can become activated, proliferate, migrate to the site of injury, and differentiate into mature neural cells [17]. However, injury-induced PVZ changes, including NPC activation, are insufficient to induce adequate structural repair or functional recovery [17]. 

In animal models, the first region of the hypothalamus (a conglomeration of nuclei located symmetrically around the 3^rd^ ventricle [18]) reported to be involved in adult neurogenesis is the mediobasal hypothalamus (MBH) [8,16]. This hypothalamic region remains the most studied, possibly due to the central role of the MBH in feeding and energy metabolism [8,16,19]. In spite of this knowledge, the comprehensive structural organization, localization and extent, as well as the functional role of the postnatal/adult hypothalamic neurogenic niche remain unknown [16]. 

This review focuses on postnatal/adult hypothalamic neurogenesis. We first introduce general aspects of neurogenesis in the embryonic and postnatal/adult CNS. We then describe the structural and functional organization of the hypothalamus and other relevant anatomical loci and discuss evidence on postnatal/adult hypothalamic neurogenesis in animal models and the postmortem human brain. Finally, we propose a “proof of concept” model of the structural and functional organization of the hypothalamic niche. In this review, the terms “postnatal” and “adult” are used interchangeably, if not otherwise specified.

## 2. NPCs in the Embryonic and Postnatal/Adult Brain

### 2.1. NPCs in the Embryonic Brain

During early embryonic development, neuroepithelial cells arise, initially forming the walls of the neural tube [20]. Neuroepithelial cells are bipolar cells of ectodermal origin, showing radial alignment with one process contacting the lumen of the ventricle (developing from the neural tube) and a second process touching the pia matter (a derivative of the neural crest) [1,4,20]. 

During brain development, the embryonic ventricular zone (VZ) is the first-appearing proliferative zone [1,20]. The VZ consists of a pseudostratified neuroepithelium that lines the ventricular system and contains NPCs, which generate the majority of the excitatory neurons in the adult neocortex, and ependymal cells [21,22,23]. During early development, neuroepithelial cells divide primarily symmetrically, expanding their number and forming the neural plate [1,3]. Following the closure of the neural tube, neuroepithelial cells begin expressing glia-specific factors, a process that delineates the differentiation of neuroepithelial cells to radial glial cells (RGCs) [1,24]. RGCs express the transcription factor Paired Box 6 (PAX6) [25]; the structural hallmark of these cells is the presence of an elongated basal process that contacts the pia matter [1].

Initially, RGCs divide symmetrically expanding their number in the VZ [1,20,21,26,27]. During cortical neurogenesis, RGCs increasingly switch to asymmetric divisions, whereby one RGC remains in the VZ while the other more differentiated daughter cell migrates distally to the overlying layers of differentiated neurons toward the cerebral cortex; thus, layers of differentiated cells arise in the forebrain [1,3,21,28]. The majority of differentiated daughter cells, which are generated by asymmetric divisions of RGCs, initially migrate to the SVZ, a secondary proliferative zone lying adjacent to the VZ [1,29,30]. In the SVZ, differentiated cells divide symmetrically, expanding their number by generating two differentiated daughter cells that then migrate to the cerebral cortex [1,29,30]. These mitotic cells are termed “intermediate progenitor cells (IPCs)”, are multipolar, and express the transcription factor T-box Brain Protein 2 (TBR2) [29,31]; this process is known as “amplification of NPC proliferation by IPCs” [32]. Similar patterns of amplifying divisions have also been described in the embryonic ventral forebrain and the adult neurogenic niches [33,34]. Toward the end of the cortical neurogenic period, RGCs translocate from the ventricles to the surface of the pia matter [1]. These cells express Glial fibrillary acid protein (GFAP) and PAX6 and, thus, they have the potential to generate both glial cells and neurons [1].

At early embryonic stages, NPCs across different CNS regions are fundamentally similar [1]. For instance, NPCs in the developing pineal gland initially express PAX6, thus resembling cortical NPCs [1,35]. By contrast, at late developmental stages, the former cells no longer resemble the latter [1,35]. In human embryos, neurogenesis starts at ~gestational week (gw) 5 and continues until newly generated neurons have migrated above the VZ toward the pial surface (at ~gw 20); neurons settle in the subplate (SP) and cortical plate (CP), while RGCs continue to touch the ventricular and pial surfaces, serving as guides for neuronal migration [36,37]. In the human developing brain, the greatest part of neurogenesis has already occurred by gw 20 [36,37]. After that, SVZ RGCs switch to gliogenesis [38]. The SVZ continues to be present during all stages of embryonic development and, in some, regions postnatally [4].

### 2.2. NPCs in the Postnatal/Adult Brain

In the postnatal/adult proliferative zones, NPCs originate from embryonic NPCs [1]. Postnatally, there is a decline in the rate of neurogenesis, with adult NPCs progressively being restricted to the adult neurogenic niches [39,40]. In the SVZ, adult NPCs constitute a subpopulation of RGCs termed “B cells”, whereas, in the SGZ, the main type of NPCs are radial-like glial cells (RLGCs) termed “type-1 cells” [1]. 

The embryonic VZ pseudostratified neuroepithelium transforms into a mixed epithelium in the adult mouse ventricular-subventricular zone (V-SVZ), with a characteristic pinwheel organization [23,41]. This mixed epithelium contains multiciliated ependymal cells (E cells) and bipolar GFAP^+^ astrocytes (B cells) [23,41]. During late embryonic and early postnatal development, distinct subpopulations of RGCs either expand their apical domains to form multiciliated ependymal cells (E cells), which are mainly distributed in the periphery of the pinwheels, or retain small apical domains to form B cells, coalescing into the centers of the pinwheels [23]. Of note, individual RGCs can generate clones of both E and B cells [23]. B cells remain mostly quiescent; however, quiescent and actively self-renewing B cells are essentially present simultaneously in the SVZ [1,42,43]. Proliferating B cells divide asymmetrically, generating B cells and IPCs, termed “C cells” [1]. C cells subsequently divide symmetrically, generating two daughter cells (termed “A cells”), which, in turn, migrate to the OB [1]. B cells express GFAP, Glutamate-Aspartate Transporter (GLAST) and Brain Lipid-Binding Protein (BLBP) [1,9,44,45]. C cells express Achaete-Scute Family BHLH Transcription Factor 1 (ASCL1) and Distal-Less Homeobox 2 (DLX2) [1,9,44,45]. A-cells express doublecortin (DCX) and PSA-NCAM [1,9,44,45]. 

In the SGZ, type-1 cells can be quiescent or active and can divide symmetrically and/or asymmetrically [1]. Quiescent type-1 cells can transform into active self-renewing adult NPCs, giving rise to newly generated neurons, which are primarily glutamatergic excitatory granule cells [1,12]. Type-1 cells express nestin, GFAP and SRY-box transcription factor 2 (SOX2), and have a radial process that projects through the granule cell layer to the molecular layer, where the endfeet of type-1 cells are in contact with synapses and the vasculature [46,47]. Type-1 cells can generate IPCs (termed “type-2 cells”), which are multipolar and express TBR2 similarly to the IPCs in the developing embryonic cortex [1]. Type-2 cells undergo a limited number of divisions, generating neuronal daughter cells that express DCX [1]. These cells then migrate radially to the granular cell layer, where they mature into Prospero Homeobox 1 (PROX1)^+^ dentate granule cells [1,48]. SGZ neurogenesis occurs in the adult brain in all mammalian species and has been functionally associated with cognitive and affective processes such as memory, learning and pattern recognition [1,49].

The organization of the SGZ is similar in humans and rodents [16]. By contrast, the organization of the SVZ in humans is different from that in rodents; the former shows a unique gap-and-ribbon organization that is absent in the latter [16]. In addition, in humans, SVZ-derived neuroblasts do not migrate to the OB but probably to the striatum [50,51]. Besides the SVZ and SGZ, in the developing retina and cerebellum, NPCs differentiate into distinct types of RGCs (retinal Müller glia and cerebellar Bergmann glia), which persist into adulthood [1]. 

Postnatal/adult NPCs are fundamentally different from embryonic NPCs [4,52]. The former are spatiotemporally restricted, show decreased plasticity and limited potency, have a longer cell cycle with a prominent quiescent phase, and have a different transcriptomic profile from the latter [4,52]. The majority of adult NPCs are in quiescence most of the time [52]. Quiescence is essentially a reversible cell cycle (in the G0 or G2 phase) arrest state, which is essential for the maintenance of the genomic integrity and functionality of NPCs [53,54]. During quiescence, RNA and protein synthesis is low and there is no expression of proliferation markers [53,54]. Nonetheless, upon stimulation, quiescent NPCs can become activated, proceeding to the next phase of the cell cycle [54].

It is unknown whether homeostasis is maintained primarily by asymmetric or symmetric divisions of adult stem cells and which mechanisms regulate the transition between quiescent and activated NPCs [3,52]. Although, in basal conditions, most adult stem cells divide asymmetrically, they maintain their capacity to divide symmetrically [3]. For example, following injury or disease, adult stem cells can revert to symmetric divisions in an effort to restore their number [3]. Of note, following stroke, the rate of cellular divisions (including symmetric) increases, enhancing neurogenesis; however, the absolute number of NPCs tends to remain unchanged, preserving homeostasis [55].

### 2.3. The Secretome of NPCs

During prenatal CNS development, NPCs synthesize and release various bioactive molecules, including morphogens (such as Sonic Hedgehog (Shh)), growth factors (such as vascular endothelial growth factor (VEGF) and platelet-derived growth factor (PDGF)), proteoglycans, apolipoprotein E (ApoE), cytokines and chemokines [56,57]. In addition, embryonic NPCs express the pleiotropic neuropeptide Pituitary Adenylate Cyclase-Activating Polypeptide (PACAP), which probably regulates temporally the switch from neurogenesis to gliogenesis [56,57]. There is sparse evidence about the secretome of NPCs during the developmental period from birth to adulthood; during this period, the secretome of NPCs is characterized by the increased expression of genes related to the regulation of the extracellular environment [56,58]. 

In the adult CNS, morphogens, growth factors, neurotrophins and cytokines regulate the proliferation and maintenance of adult NPCs and newly differentiated neurons, in an autocrine or/and paracrine fashion [56]. In essence, most of these signaling and neurotrophic molecules simulate their effects in ESCs; however, the cellular microenvironment in the adult brain is different from that in the developing brain [56]. In the SVZ and SGZ, Shh is essential for the maintenance of quiescence and, thus, of the pool of NPCs [56]. Interestingly, in the SGZ, RGLCs coexpress Shh and SOX2, with the expression of the former depending on the presence of the latter [59]. SGZ RGLCs also express Wnt, a pleiotropic molecule involved in the maintenance of quiescence and self-renewal but also the differentiation of NPCs [60,61]. In addition, SGZ RGLCs express IGF2, VEGF, Milk Fat Globule-EGF Factor 8 protein (MFGE8), glycosylated cystatin C, and Stem cell-Derived Neural stem/progenitor cell Supporting Factor (SDNSF); these molecules act in an autocrine manner, increasing the survival of NPCs [56]. Adult NPCs express the nuclear receptor/transcription factor TLX (encoded by NR2E1), which plays a key role in the proliferation and maintenance of the undifferentiated state of NPCs, as well as in the differentiation of RGCs into astrocyte-like NPCs [62,63,64]. Of note, TLX knockout animals show a reduced rate of NPC proliferation, and this effect is associated with impaired spatial learning, indicating that adult NPCs may be functional [64]. 

Initially, it was thought that structural cell replacement was the mechanism through which transplanted NPCs were beneficial to the host nervous system [57,65]. However, there is evidence that NPCs can signal to endogenous host cells through their secretome, i.e., through the release of neurotrophic and neuroimmunomodulatory factors, as well as of extracellular vesicles (EVs) (vide infra), ultimately being pro-regenerative [57,65].

#### EVs and NPCs

EVs are bilayer membrane-enclosed nanoparticles (mainly exosomes and microvesicles), which are produced and released into the extracellular space from virtually all cell types including mature neural cells and NPCs [66]. EVs can be taken up by neighboring or distant target cells, acting in an autocrine, paracrine and/or endocrine fashion [66]. EVs contain bioactive molecules such as nucleic acids, proteins and lipids, and are present in biofluids such as the blood and CSF [66].

Embryonic NPC-derived EVs can be internalized by neighboring astrocytes and NPCs, inducing the differentiation of the latter into neurons and glial cells [67]. These EVs are enriched with miR-9, a microRNA (miRNA) with a key role in the determination of neural fate and synaptic morphology [68]. miR-9 is transferred to neighboring NPCs, where it targets the Hes Family BHLH Transcription Factor (Hes1), regulating neurogenesis and gliogenesis [68,69]. The inhibition of the synthesis and release of exosomes impairs the maintenance of the quiescence of NPCs, as well as their transition from the active to the quiescent state [70]. Hence, the synthesis and release of exosomes might be a means of regulating the quiescent state of NPCs, through yet unknown mechanisms [70]. 

In adult mice, EVs derived from SVZ NPCs can deliver functional mitochondria to target cells, potentially restoring mitochondrial dysfunction [71]. In the neonatal SVZ, NPCs release EVs that can be internalized by microglia, altering microglial morphology and increasing the microglial expression of interleukin (IL) 1-alpha (IL-1α), IL-1β and IL-6 [72]. NPC-derived EVs carry miRNAs, including let-7, miR-9, miR-26 and miR-181, among which let-7 is the most abundant [72]. NPC-derived exosomal let-7 activates microglia through the stimulation of endosomal Toll-like receptor 7 (TLR7), for which let-7 acts as a ligand [72]. Activated microglia then inhibit the proliferation of NPCs, forming a negative feedback loop [72]. Thus, NPC-derived EVs may serve as noncanonical morphogens, through microglial activation [72]. 

The mechanism through which transplanted adult stem cells communicate with host immune cells may be cellular signaling mediated by interferon gamma (IFNγ)/IFNγ receptor 1 (IFNGR1) complexes originating from adult stem cell EVs [73]. Adult mouse SVZ NPCs treated with pro-inflammatory cytokines release EVs that are enriched with IFNγ pathway-related mRNAs [73]. IFNγ binding to IFNGR1 on the surface of EVs induces the activation of the Signal Transducer and Activator of Transcription 1 (STAT1) in target cells [73]. This effect may be mediated through the binding of IFNγ (dissociated from source cell-derived EV IFNGR1s) to IFNGR1s in target cells, triggering the IFN/STAT1 pathway in the latter [73]. 

In an in vitro hypoxia/reperfusion injury model, coculture of human NPC-derived EVs with neurons inhibits the apoptosis of the latter by inducing the translocation of NF-E2-related factor-2 (NRF2) to neuronal nuclei, regulating the expression of oxidative stress-induced kinases [74]. Interestingly, coculture of such exosomes with human umbilical vein endothelial cells (HUVECs) enhances angiogenesis in the latter [74]. Bilateral cranial transplantation of human NPCs or NPC-derived EVs decreases the effect of cranial irradiation on dendritic complexity and spine density in the ipsilateral and contralateral hippocampi, and prevents microglial activation; these effects are associated with the upregulation of Glial cell line-Derived Growth Factor (GDGF) and the rescue of the irradiation-induced increase of Postsynaptic Density Protein 95 (PSD95) [75]. Intranasal administration of ihPSC-derived NPC EVs promotes neurogenesis in the intact adult brain and exerts anti-inflammatory effects in the injured adult brain [76]. In virtually all adult brain regions, EVs can be rapidly internalized by neurons, microglia and some astrocytes [76]. In male rats, intravenous administration of NPC-derived EVs immediately after traumatic brain injury (TBI) results in a significant reduction in the size of TBI lesions, and this effect is associated with an increase in the number of endogenous NPCs [77]. NPC-derived EVs have neuroprotective effects in vitro and in vivo, decreasing the levels of ROS and pro-inflammatory cytokines, thus inhibiting apoptosis and neuroinflammation, which are the hallmarks of Parkinson’s disease (PD) [78]. NPC-derived EVs are enriched with miRNAs involved in neurogenesis, cell differentiation and the immune response, such as miR-17, miR-20a-5p, miR-182 and miR-183 [78].

## 3. Regulation of Adult Neurogenesis

### 3.1. Regulation of Adult Neurogenesis by Local and/or Distal Neural Circuits

In contrast to embryonic neurogenesis, pre-existing local and distant neural circuits, influenced by experience, dynamically regulate adult neurogenesis through yet elusive mechanisms [79].

#### 3.1.1. Neurotransmitters

Neurotransmitter signaling via GABA, acetylcholine, glutamate, serotonin and dopamine influences adult neurogenesis, at least in the traditional neurogenic niches [79,80,81,82,83,84]. 

Distal GABAergic and cholinergic neurons regulate adult DG neurogenesis directly and/or indirectly [79]. The DG receives GABAergic and cholinergic projections from the basal forebrain; the former end at GABAergic interneurons, whereas the latter are excitatory, ending primarily at DG granule cells [79]. In addition, quiescent RGLCs receive activity-dependent GABAergic input from local parvalbumin (PV)^+^ interneurons, through the activation of RGLC GABA_A_Rs by GABA that spills over from PV^+^ interneuron − mature granule cell synapses [79,85,86]. Of note, when DG neuronal activity is high, activated PV^+^ interneurons inhibit the activation of quiescent NPCs, while, at the same time, they enhance the survival of proliferating (active) NPCs [79]. By contrast, when DG neuronal activity is low, the inhibition of PV^+^ interneurons allows the expansion of the pool of quiescent NPCs, while, at the same time, the survival of proliferating NPCs is suppressed [79]. 

Newborn DG granule cells receive glutamatergic input from the entorhinal cortex [79]. In addition, these neurons depend on local NMDAR-mediated signaling through transient local glutamatergic inputs from mature granule cells to their immature counterparts [79,80,84]. 

The DG (especially the SGZ) receives dense serotoninergic input from the raphe nuclei, which modulates local GABAergic interneurons that influence DG neurogenesis [79]. Overall, increased serotonin levels promote the proliferation and differentiation of DG NPCs, whereas serotonin depletion inhibits NPC proliferation and differentiation [87,88]. Nonetheless, the 5-HT receptor subtype (activated by serotonin) determines the effects of serotonin on DG neurogenesis [79]. 

The DG receives diffuse dopaminergic projections mainly from the ventral tegmental area (VTA) [79]. Dopamine influences the proliferation of SGZ NPCs through the activation of different receptor subtypes as well as through the modulation of local GABAergic interneurons, thus fine-tuning the excitation/inhibition balance [79]. The role of dopamine in adult neurogenesis remains controversial [79]. 

Similarly to the DG, SVZ neurogenesis can be regulated directly or indirectly by local GABAergic interneurons or distal GABAergic projections to the SVZ [79,89,90]. GABA significantly influences various stages of SZV/OB neurogenesis, including the proliferation of astrocyte-like NPCs and neuroblasts, as well as neuroblast differentiation and migration [79]. Interestingly, neuroblasts themselves release GABA, thus regulating other more immature forms of NPCs [79]. In addition, migrating neuroblasts transiently contact local mature neurons; thus, in the SVZ, newly generated neurons might first receive direct input from local GABAergic neurons, similarly to the SGZ [84].

In the rat adult OB, virtually all newly generated neurons become local interneurons; the majority of them differentiate into granule cells [79]. Of note, the first synapses of granule cells are with proximally-originating excitatory glutamatergic neurons, just a few days after newly generated neurons reach the OB; however, the origin of this glutamatergic input remains unknown [79,84]. 

The SVZ receives serotoninergic input from the raphe nuclei [79]. Generally, serotonin promotes SVZ neurogenesis; however, the serotoninergic effect depends on the stimulated 5-HT receptor subtype [79,91]. 

The SVZ receives dopaminergic input from the substantia nigra [80]. Dopamine receptor D2 and D3 (D2R and D3R) agonism increases the number of proliferating SVZ NPCs and promotes the maturation and differentiation of SVZ/OB NPCs into neurons [79,92].

Essentially, local (adjacent to the adult neurogenic niches) and distal neural circuits can influence NPCs and their progeny directly or indirectly [79]. As local and distant networks interact extensively at various levels, it is plausible that the complex interactions between different neural networks and neuromodulatory systems (modulated by environmental and experiential stimuli) collectively fine-tune adult neurogenesis, at least in the “traditional” neurogenic niches [79]. 

#### 3.1.2. Neuropeptides

In the adult DG, Vasoactive Intestinal Peptide (VIP) coreleased with GABA from GABAergic interneurons enhances the proliferation of nestin^+^ NPCs via the activation of VIP receptor 2 (VPAC2), shifting the fate of mitotically dividing NPCs toward a nestin-only phenotype [93]. On the other hand, activation of VIP receptor 1 (VPAC1) shifts the NPC fate toward a granule cell phenotype [93]. In the DG, Neuropeptide Y (NPY) is released selectively from GABAergic interneurons, increasing neurogenesis through the activation of Y1R [94,95,96]. In the SVZ, most NPY originates from the CSF [95]. In addition, NPY is released from subsets of SVZ subependymal cells and NPCs, acting in an autocrine and paracrine fashion, through the activation of Y1R primarily expressed in SOX2^+^/nestin^+^ cells and DCX^+^ neuroblasts [95,96]. PACAP receptor 1 (PAC1) is expressed in the SVZ and SGZ [97]. The activation of PAC1 by PACAP potently induces the proliferation of NPCs [97]. Interestingly, neuropeptides can diffuse over a relatively large distance from the point of their release, reaching and activating distant targets through volume transmission [98]; thus, neuropeptide-releasing neuronal projections originating from local or distal circuits may influence the neurogenic niches.

### 3.2. The Effects of Glucocorticoids (GCs) on Adult Neurogenesis

GCs bind both the glucocorticoid receptor (GR) and mineralocorticoid receptor (MR), although with different affinity (low and high, respectively) [99]. In the human hippocampus, GR activation decreases the proliferation of NPCs and their differentiation into neurons but does not affect the differentiation of NPCs into astrocytes [99]. On the other hand, MR activation promotes the proliferation of NPCs and their differentiation into astrocytes but suppresses the NPC differentiation into neurons [99]. Hence, low-dose GCs may exert their effects through the MR (thus, promoting neurogenesis), whereas high-dose GCs may mediate their effects through the GR (thus, suppressing neurogenesis) [99]. Low-dose GCs activate the Notch/Hes pathway, promoting the proliferation of neural progenitors and their switch to an astroglial cell fate [99,100]. On the other hand, high-dose GCs inhibit the Notch/Hes and TGFβ-SMAD2/3 pathways and activate FOXO3A [99]. Notch/Hes signaling suppression may inhibit the MR activation-induced effects on NPC proliferation and astrogliogenesis [99]. On the other hand, TGFβ signaling promotes neurogenesis; thus, inhibition of TGFβ may contribute to the GR activation-dependent decrease in neurogenesis [99,101,102]. Of note, both GC doses inhibit the Hedgehog pathway [99,103,104].

## 4. Markers of Adult Neurogenesis

SOX2 is a member of the family of SRY-box transcription factors (that contain a DNA-binding domain (high-mobility group, HMG)) [105]. SOX2 is expressed in proliferating NPCs and cells with stem cell-like characteristics [106]. SOX2 interacts and forms a complex with Octamer-binding Transcription Factor 4 (OCT4) (a cofactor); this complex mediates the recruitment of other nuclear factors, activating the expression of genes related to pluripotency while repressing genes involved in differentiation [107]. Therefore, SOX2 is a transcriptional modulator that imposes cell fate-determining expression patterns [108]. SOX2 regulates the expression of Fibroblast Growth Factor 2 (FGF2) and nestin [105,109].

Nestin (a neuroepithelial stem cell marker) is a type-VI intermediate filament, initially described in NPCs of the developing and adult brain; however, nestin is also expressed in various non-neural tissues [110,111,112]. Nestin influences NPC self-renewal, migration and differentiation [110]. During mitosis, nestin is involved in the assembly/disassembly of other intermediate filaments, such as vimentin [113]. In addition, nestin acts as a mediator of the interaction of intermediate filaments with microtubules and microfilaments [114]. However, the pathways that control nestin expression and, thus, function remain unknown [110].

Vimentin is a type-III intermediate filament protein used as a marker of RGCs [115]. During the transition of NPCs from quiescence to activation, NPCs form aggresomes (intracellular aggregations of misfolded proteins into a single location) as a mechanism of clearing these proteins when the degradation system of the cell (proteasome) is overwhelmed [115,116]. During aggresome formation, vimentin is redistributed to a single pericentriolar site [116]. During the exit of NPCs from quiescence, vimentin organizes protein turnover at the aggresome [117]. 

GFAP (a type-III intermediate filament protein) and the glutamate transporter GLAST are markers of astroglial or astroglia-like cells, including RGCs [118,119,120,121]. GFAP is present in NPCs, astrocytes (CNS), non-myelinating Schwann cells (PNS), and enteric glial cells [119,120]. Interestingly, primary astrocyte cultures contain GFAP-expressing cells that can act as multipotent NPCs when transferred to neurogenic conditions [122]. However, GFAP-expressing NPCs are phenotypically and functionally distinct from non-neurogenic astrocytes [122]. During CNS development, GLAST is expressed in astroglia-like cells across different maturational stages from RGCs through astrocytes [123]. GLAST^+^ RGCs may be intrinsically different from nestin^+^ RGCs; the former are responsible for long-term neurogenesis, whereas the latter contribute to short-term neurogenesis [123].

BLBP is a brain-specific member of the lipid-binding protein family; these proteins transfer small hydrophobic signaling molecules between cellular compartments [124]. In the CNS, BLPB is exclusively expressed in RGCs and immature astrocytes, whereas in the PNS, BLBP is also expressed in glial cells [124,125,126]. In the mouse brain, almost all neuronal subpopulations originate from BLBP^+^/GLAST^+^ RGCs, which serve as progenitors for most CNS neurons, after a spatiotemporally patterned neurogenic stage [127]. Of note, RGCs in the ventral telencephalon complete this stage earlier than RGCs in the dorsal telencephalon [127]. BLPB/GLAST expression is absent in neuroepithelial cells; however, it is a marker of the maturation of neuroepithelial cells to RGCs, heralding the onset of radial glia neurogenesis [127,128,129].

PAX6 is a highly conserved transcription factor that targets numerous NPC promoter sites, activating ectodermal genes (including these encoding other transcription factors that are critical for neurogenesis, such as NFIα and TBR2) and, at the same time, repressing mesodermal and endodermal genes, ensuring the unidirectionality of lineage commitment toward neuronal differentiation [130]. Many of these promoters are co-targeted by SOX2, indicating that PAX6 and SOX2 are members of the same gene regulatory network [130].

Mushasi proteins (MSI) are highly conserved RNA-binding proteins that upregulate Notch signaling [131]. MSI1 is expressed in the somata of astroglia-like NPCs (including RGCs) located in the periventricular areas of the embryonic and adult brain (in rodents and humans), as well as in mature GFAP^+^ astrocytes [129,131]. MSI1 is not expressed in OPCs [131].

The Proliferating Cell Nuclear Antigen (PCNA) is involved in DNA replication and repair [132]. PCNA is upregulated during the G1 and S phases of the cell cycle and downregulated during the cell transition into the G2 and M phases; however, PCNA can also be detected in the early G0 phase [133]. PCNA is expressed in a subgroup of SVZ and SGZ actively proliferating NPCs, and thus has been used as a proliferation marker [134]. 

Ki67 is a nuclear protein used as a marker of dividing cells [135]. Ki67 is expressed during all phases of the cell cycle except for the G0 and early G1 phase [135]. Anti-Ki67 antibodies yield less variable results than those achieved with anti-PCNA [135]. Hence, Ki-67 may be a more specific marker of proliferation than PCNA [135]. 

MCM2 is expressed specifically in the G1 phase of the cell cycle [136]. MCM2 expression can be used as a marker of cells resting in the G1 phase between cell divisions, such as slowly cycling NPCs and rapidly cycling proliferative NPCs [136].

PSA-NCAM (the polysialylated form of the neural cell adhesion molecule) is a marker of young migrating neurons in the postnatal/adult mammalian brain [137]. However, the most widely used surrogate marker of newborn migrating neurons is DCX, a protein involved in the structure of microtubules, the latter participating in the radial and tangential migration of neuroblasts in the developing brain [138]. DCX is expressed in late neural progenitors (immature neurons) but is downregulated after their full differentiation/maturation [139]. DCX is upregulated in regions of adult neurogenesis, including the SVZ, SGZ and OB [140]. Of note, expression of PSA-NCAM in conjunction with DCX further supports the neurogenic potential of DCX+ cells, whereas expression of HuC/D (a marker of young neurons) reflects more mature neuronal phenotypes [140].

Bromodeoxyuridine (BrdU), a thymidine analog, is an exogenous cell tracer incorporated into dividing cells during the S phase of the cell cycle [141]. After its incorporation, BrdU remains in place and is passed down to daughter cells [141]. BrdU can be detected by a monoclonal antibody against BrdU-containing single-stranded DNA (immunochemistry) [142]. BrdU has been considered a marker of DNA synthesis and cell proliferation in developmental neuroscience and adult neurogenesis studies [143]. Nonetheless, BrdU is also incorporated into cells undergoing DNA repair, abortive cell cycle re-entry initiating apoptosis, and gene duplication without cell division, the latter being a hallmark of polyploidy [143]. Therefore, BrdU is only a marker of DNA synthesis, not of the S phase of the cell cycle [143]. In addition, BrdU can be transferred from dying cells to neighboring dividing cells [143]. BrdU is mutagenic and toxic, altering DNA stability and the cell cycle and potentially leading to cell death [143]. Hence, BrdU immunoreactivity should be interpreted with caution and alternative methods should be considered for the study of newly generated neurons in the mammalian brain [143].

NeuN, a highly conserved nuclear protein across species, has traditionally been used as an exclusive marker of postmitotic, mature neurons (anti-NeuN antibody) [144]. NeuN is expressed in neuronal precursors only after their migration [144]. NeuN is an epitope of the RNA Binding Fox-1 Homolog 3 (RBFOX3), a member of the RBFOX1 family of splicing factors [145,146]. NeuN/RBFOX3 is expressed in the nuclei of mature neurons, in virtually all parts of the vertebrate nervous system [144,147,148,149]. However, some neuronal cell subtypes, including Purkinje cells, OB mitral cells, Cajal-Retzius cells, neurons in the inferior olivary nucleus, dentate nucleus, sympathetic ganglia, retinal photoreceptor cells, inner nuclear layer, and a proportion of SCN neurons, cannot be labeled for NeuN [144]. In addition, NeuN immunoreactivity is variable in dopaminergic neurons and is lost in the cervical, thoracic and lumbar segments of aged rats [144,150]. Negative NeuN immunoreactivity does not necessarily correspond to neuronal loss in several pathophysiological conditions, including stroke, PD, tuberous sclerosis, the irradiated hippocampus, and the aged spinal cord [150,151,152,153,154]. Thus, NeuN immunoreactivity should be interpreted with caution [144].

## 5. The Hypothalamus

**Figure 1:**
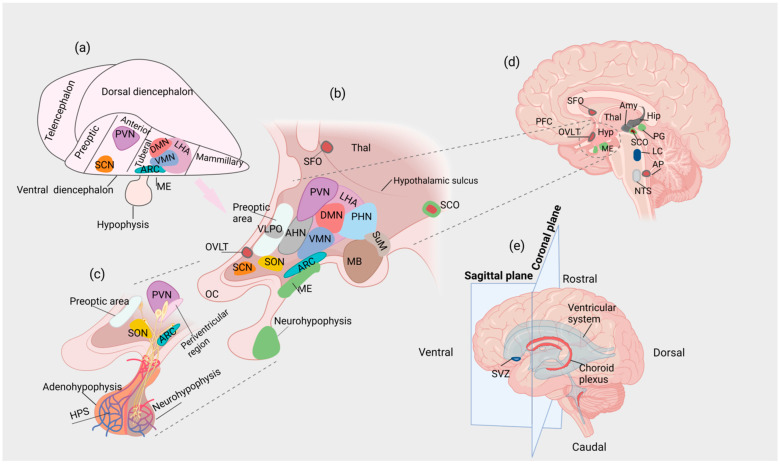
(**a**) Lateral view of the vertebrate forebrain showing the conserved hypothalamic subdivisions after initial patterning. (**b**) Organization of the hypothalamic nuclei in the adult human brain (sagittal plane). (**c**) Parvocellular neurons end at the HPS, whereas magnocellular neurons project to the neurohypophysis. (**d**) Sagittal section of the adult human brain at the level of the hypothalamus. Secretory CVOs are depicted in light green, whereas sensory CVOs are depicted in deep red. The SCO is considered a secretory and sensory CVO (**e**) Directions and planes of section that determine the localization of brain structures and anatomical subdivisions. Amy: amygdala; AP: area postrema; ARC: arcuate nucleus; CVO: circumventricular organ; DMN: dorsomedial nucleus; Hip: hippocampus; HPS: hypophyseal portal system; Hyp: hypothalamus; LC: locus caeruleus; LHA: lateral hypothalamic area; MB: mammillary body; ME: median eminence; NTS: nucleus tractus solitarius; OC: optic chiasm; OVLT: organum vasculosum of the lamina terminalis; PFC: prefrontal cortex; PG: pineal gland; PHN: posterior hypothalamic nucleus; PVN: paraventricular nucleus of the hypothalamus; SCN: suprachiasmatic nucleus; SCO: subcommissural organ; SFO; subfornical organ; SON: supraoptic nucleus; SuM: supramammillary nucleus; SVZ: subventricular zone; Thal: thalamus; VMN: ventromedial nucleus.

The hypothalamus is a brain region that integrates signals from the periphery of the organism and the external environment, regulating core physiological processes, including food intake, energy metabolism, growth, reproduction, sleep and aging, as well as the neuroendocrine stress response, ultimately aiming at the maintenance of organismal homeostasis [66,155,156,157]. The hypothalamus consists of multiple nuclei (i.e., agglomerations of neurons) organized in a 3D network around a small area of the ventral portion of the 3^rd^ ventricle [18,157,158] (Figure 1).

A proportion of these neurons are specialized neuroendocrine (neurosecretory) cells that release a variety of peptides into the blood vessels of the hypophyseal portal system (HPS) or the systemic circulation [18,66]. Hypothalamic neuroendocrine cells receive “classical” neurotransmitter input from neurons originating from various non-hypothalamic CNS regions, allowing neuroendocrine integration to occur [66]. At the base of the 3^rd^ ventricle and within the hypothalamus, the median eminence (ME) is a circumventricular organ (CVO), i.e., a site in which the BBB is incomplete [159]. The hypothalamic nuclei show relative specialization, regulating distinct functions (e.g., the suprachiasmatic nucleus (SCN) regulates circadian rhythms) [18,160]. However, due to the reciprocal interactions between different hypothalamic nuclei at various levels, there might be functional overlaps, i.e., distinct hypothalamic nuclei may contribute to the regulation of the same physiological process and vice versa.

Two types of neuroendocrine cells (parvocellular and magnocellular neurons) regulate the control of hypophyseal hormones [18]. The paraventricular nucleus of the hypothalamus (PVN) but also the ARC, the periventricular region, and nuclei in the pre-optic area contain the somata of the former, while the somata of the latter are located in the PVN and supraoptic nucleus (SON) [18]. Parvocellular neurons project to the external layer of the ME, where their endings terminate at the bed of the fenestrated capillaries of the HPS, releasing neuropeptides, which are transported to the cells of the anterior hypophysis (adenohypophysis), stimulating or inhibiting the secretion of hypophyseal hormones [18]. On the other hand, magnocellular neurons project to the posterior hypophysis (neurohypophysis), releasing arginine vasopressin (AVP) and oxytocin (Oxt) directly into the systemic circulation [18,161]. PVN parvocellular neurons release primarily corticotropin-releasing hormone (CRH) and thyrotropin-releasing hormone (TRH), whereas parvocellular neurons in the periventricular region, preoptic area and ARC secrete somatostatin; gonadotropin-releasing hormone (GnRH); and dopamine, GnRH and growth hormone-releasing hormone (GHRH), respectively [162,163]. Neuraxons of magnocellular AVP-producing neurons transverse the internal layer of the ME, projecting to the neurohypophysis, where AVP is released into the systemic circulation from their axon terminals [164]. In the PVN and SON, the synthesis of AVP is regulated by osmotic pressure and/or body fluid volume [164,165]. Although AVP is also produced in the dorsomedial part of the SCN, AVP-producing SCN neurons are not considered part of the neuroendocrine system [164]. It has been proposed that AVP released from the SCN is involved in the regulation of biological rhythms; however, the comprehensive physiological roles of AVP^SCN^ are still under investigation [166,167]. CRH and AVP stimulate individually and synergistically the release of adrenocorticotropic hormone (ACTH) from the adenohypophysis [168]. Of note, the concentration of AVP in the hypophyseal portal capillaries increases when animals are stressed or have undergone adrenalectomy [169,170,171]. Another subpopulation of AVP-producing neurons is located in the posterolateral part of the PVN [172]. These neurons project to extrahypothalamic regions of the brain, as well as the spinal cord, probably involved in the regulation of autonomic output [172].

The anatomical and functional organization of the hypothalamus, including neuronal subtypes and their relative localization, is remarkably conserved across vertebrate species as diverse as fish and mammals, probably due to the fundamental roles of the hypothalamus in the regulation of core homeostatic processes [173]. This remarkable anatomical conservation may correspond to conserved molecular mechanisms that regulate hypothalamic induction, patterning and neurogenesis [173]. Interestingly, according to evidence from comparative studies, species-specific developmental programs that associate anatomy, cellular differentiation and gene expression may exist, resulting in the generation of “modules” that may be either preserved or lost throughout evolution [173]. For example, in zebrafish, continuous neurogenesis occurs through a posterior ventricular recess, which is absent in mammals [174,175]. Of note, several neuronal subtypes that are present in this region in non-mammalian vertebrates (e.g., histaminergic neurons) are also present in the premammillary region of the hypothalamus in rodents, indicating anatomical and functional homology [173,176]. By contrast, dopaminergic neurons expressing Tyrosine Hydroxylase 2 (TH2), present in the posterior ventricular recess in zebrafish, are absent in mammals, in which the TH2 gene has been lost [177,178]. The above findings indicate that, during evolution, the neuronal networks that are present in the zebrafish posterior ventricular recess might have translocated to other brain regions, or these functions may no longer be necessary for mammalian organisms; thus, they may have been abolished due to lack of survival value [173]. After initial patterning, the conserved hypothalamic subdivisions in a vertebrate prototype model comprise four regions: (i) preoptic, (ii) anterior, (iii) tuberal and (iv) mammillary; each region consists of groups of nuclei with associated functions [173,179] (Figure 1a).

During the specification of the neural plate, the region destined to develop into the hypothalamus is located in the midline and rostrally, in close contact with the future hypophysis, which, at this stage, is present in the form of the hypophyseal placode [155,173,180]. Increased proliferation of NPCs in the dorsal telencephalon—compared to the ventral telencephalon—results in a shift of the prospective hypothalamus posteriorly and ventrally to the telencephalic ventricles [155]. At the midline, the infundibulum arises as a local extension of the neuroepithelium toward the developing hypophysis, connecting the ME to the latter [155]. As expected, infundibular NPCs have the potential to generate both neurons and glial cells [181,182,183]. Shh signaling is vital for early hypothalamic specification and subsequent regionalization [184,185,186,187]. By contrast, the specification of the infundibulum is determined by the antagonism between Shh and members of the Bone Morphogenetic Protein family (BMP), while members of the Fibroblast Growth Factor (FGF) Family are essential for the expansion of the number of infundibular cells [181,186,187]. In addition, Notch signaling is necessary for the formation of the infundibulum; deletion of HES1 and HES5 (effectors of Notch) results in the premature exit from the cell cycle and inefficient evagination of the ventral diencephalon, leading to complete loss of the posterior hypophyseal lobe, while NPCs in the ventral diencephalon differentiate into neurons at the expense of pituicytes [183]. The expression of LIM Homeobox 2 (LHX2) and T-Box Transcription Factor 3 (TBX3) is also crucial for proper infundibular morphogenesis, which in turn is essential for the induction and maintenance of the Rathke’s pouch and, thus, the formation of the hypophysis [186,188,189]. Loss of LHX2 and TBX3 results in cellular hyperproliferation; thus, a balance between proliferation and migration is essential [181]. 

In mammalian models, tanycytes appear in the infundibulum during late gestation, while their terminal differentiation takes place postnatally [190]. Tanycytes originate from embryonic infundibular NPCs, in congruence with adult SVZ NPCs arising from slowly dividing embryonic NPCs [24,43]. In the embryo, there is relative flexibility between tanycytic and ependymal cell fates; however, this flexibility is lost postnatally [24]. The transcription factors LHX2 and Retina And Anterior Neural Fold Homeobox Protein (RAX) are essential for ventral hypothalamic development as well as tanycytic specification and differentiation; these transcription factors are expressed in both embryonic and postnatal tanycytes [188,191,192,193]. In LHX2 knockout embryos, tanycytic specification is impaired, and ependymal cell-fate markers are upregulated [191]. In addition, the postnatal terminal differentiation of α and β tanycytes is hindered [191]. Loss of RAX results in an intermediate phenotype whereby NPCs acquire multiple cilia (a feature of ependymal cells); however, some tanycytic characteristics, such as the radial glia morphology, are maintained [191]. Of note, at early developmental stages, RAX expression regulates LHX2 in the MBH, whereas, later, there is bidirectional regulation between RAX and LHX2, while, postnatally, LHX2 is required for the maintenance of RAX expression [191]. In the postnatal brain, the number of tanycytes is regulated by Wnt signaling [175].

### 5.1. Hypothalamic Nuclei in the MBH

The MBH is known primarily for its role in the regulation of several aspects of metabolic homeostasis, including feeding behavior, body weight and glucose metabolism. The MBH comprises the ARC, DMN and VMN as well as the ME and the pars tuberalis (PT) [194].

#### 5.1.1. The ARC

**Figure 2:**
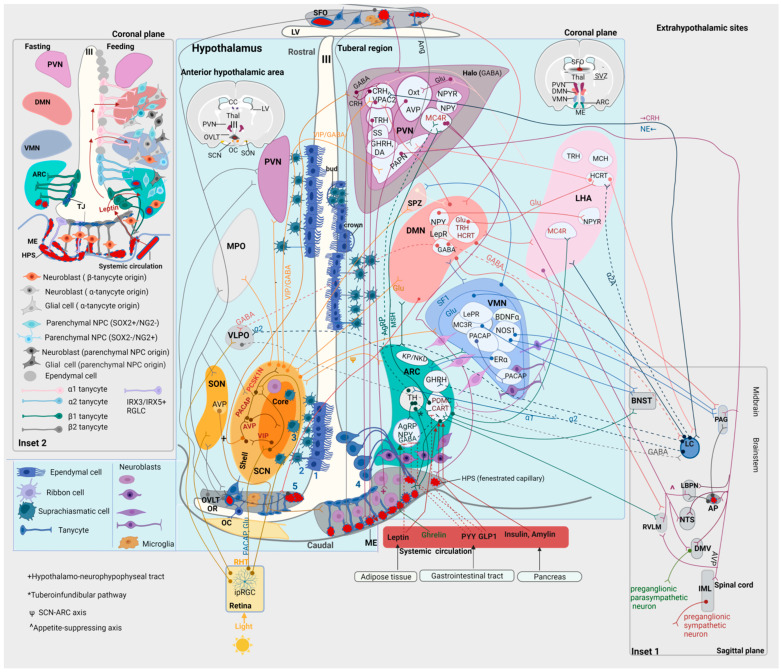
Main figure. Hypothalamic nuclei with main neuronal subpopulations and their projections at the levels of (i) the anterior hypothalamic area (on the left of the 3^rd^ ventricle, III) and (ii) the tuberal region (on the right of the 3^rd^ ventricle, III) in the human brain (coronal plane). The human hypothalamic neurogenic niche: 1: ependymal cells, 2: ribbon cells, 3: suprachiasmatic cells, 4: tanycytes, and 5: OVLT NPCs. At the bottom right, anorexigenic and orexigenic peptides from the systemic circulation reach the ARC through the ME. At the bottom left, light activates the RHT. Ependymal and ribbon cells are present throughout the rostrocaudal and dorsoventral levels of the wall of the 3^rd^ ventricle except for the ventral tuberal region, where the 3^rd^ ventricle is lined by tanycytes. The adult human hypothalamus contains DCX^+^ neuroblasts with different morphologies depending on location. Inset 1. Sagittal plane of the brainstem at the level of the AP. Projections of hypothalamic nuclei to midbrain and brainstem nuclei. Inset 2. The rodent hypothalamic neurogenic niche. Distinct subpopulations of tanycytes line the wall of the 3^rd^ ventricle. Circulating leptin is transported to the cerebrospinal fluid via transcytosis through the endfeet of tanycytes. During fasting, VEGFA and tight junction proteins are upregulated. AgRP: agouti-related peptide; Amy: amygdala; Ang: angiotensin; AP: area postrema; ARC: arcuate nucleus; AVP: arginine vasopressin; BDNF: brain-derived neurotrophic factor; BNST: bed nucleus of the stria terminalis; CART: cocaine- and amphetamine-regulated transcript; CC: corpus callosum; CRH: corticotropin-releasing hormone; DA: dopamine; DMN: dorsomedial nucleus; DMV: dorsal motor nucleus of the vagus; ERα: estrogen receptor α; GHRH: growth hormone-releasing hormone; GLP1: glucagon-like peptide-1; Glu: glutamate; HCRT: hypocretin (orexin); HPS: hypophyseal portal system; IML: intermediolateral column of the spinal cord; ipRGC: intrinsically photosensitive retinal ganglion cell; KP: kisspeptin; LHA: lateral hypothalamic area; LBPN: lateral parabrachial nucleus; LepR: leptin receptor; LV: lateral ventricle; MC4R: melanocortin 4 receptor; MCH: melanin-concentrating hormone; ME: median eminence; MPO: medial preoptic nucleus; MSH: melanocyte-stimulating hormone; NE: norepinephrine; NKD: neurokinin D; NOS: nitric oxide synthase; NPC: neural progenitor cell; NPY: neuropeptide Y; NTS: nucleus tractus solitarius; OC: optic chiasm; OR: optic recess; OVLT: organum vasculosum of the lamina terminalis; Oxt: oxytocin; PACAP: pituitary adenylate cyclase-activating polypeptide; PAC1: PACAP receptor type 1; PAG: periaqueductal gray; PAPN: preautonomic projecting neurons; PCSK1N: proprotein convertase subtilisin/kexin type 1 inhibitor; POMC: proopiomelanocortin; PVN: paraventricular nucleus of the hypothalamus; PYY: peptide YY; RHT: retinohypothalamic tract; RGLC: radial glia-like cell; RVLM: rostroventrolateral medulla; SCN: suprachiasmatic nucleus; SFO; subfornical organ; SON: supraoptic nucleus; SPZ: subparaventricular zone of the hypothalamus; SS: somatostatin; SVZ: subventricular zone; Thal: thalamus; TH; tyrosine hydroxylase; TJ: tight junction; TRH: thyrotropin-releasing hormone; VIP: vasoactive intestinal peptide; VLPO: ventrolateral preoptic nucleus; VMN: ventromedial nucleus; VPAC2: vasoactive intestinal peptide receptor 2; III: third ventricle.

The ARC extends to the ME, forming an anatomical and functional complex with the latter (ARC-ME) [195] (Figure 1 and Figure 2). The ARC contains subsets of neuropeptide- and neurotransmitter-releasing neurons; these signaling biomolecules are pleiotropically involved in a variety of physiological processes, such as the regulation of food intake and energy expenditure, prolactin release, reproduction and onset of puberty [195]. The best-studied groups of ARC neurons are (a) the TH^+^ (dopaminergic) neurons and (b) two functionally antagonistic types of neurons: the orexigenic and anorexigenic neurons; the former express NPY and Agouti-Related Peptide (AgRP), while the latter express proopiomelanocortin (POMC) and Cocaine- and Amphetamine-Regulated Transcript (CART) [196,197]. Interestingly, transcriptomic analysis of the mouse ARC-ME complex revealed the existence of ~fifty transcriptionally distinct neuronal subpopulations, including (i) three types of POMC neurons, (ii) two types of AgRP neurons, (iii) six types of dopaminergic neurons, (iv) a subpopulation of neurons expressing kisspeptin (KP) and neurokinin D (NKD), and (v) GHRH neurons [198,199] (Figure 2).

As mentioned above, the ME (as a CVO) has fenestrated capillaries; thus, it is a region of incomplete BBB [200] (Figure 2). ARC neurons are in contact with the infundibular recess at the floor of the 3^rd^ ventricle, allowing the exchange of signals between the CSF and neighboring hypothalamic structures [195]. In humans, the infundibular recess passes through the center of the pituitary stalk, connecting the 3^rd^ ventricle with the hypophysis [201]. The wall of the infundibular recess contains tanycytes [202]. 

One of the major neuronal pathways arising from the ARC is the tuberoinfundibular pathway (one of the four main dopaminergic pathways in the brain), extending from the ARC to the ME and the infundibular stem [195]. The axons of the TH^ARC^ neurons end at the loops of fenestrated capillaries of the HPS at the external layer of the ME, releasing dopamine [195] (Figure 2); this hormone binds to D2 receptors on the lactotroph cells of the adenohypophysis, inhibiting the synthesis and release of prolactin [203]. Therefore, the connection of the tuberoinfundibular tract with the HPS represents a neuroendocrine link between the hypothalamus and the adenohypophysis [204,205]. Prolactin has pleiotropic actions, including homeostatic (regulation of metabolism, fluid balance, immune function and adaptation to stressors) and reproductive (production of sex steroids, lactation, gestation and parental behavior) [204,205]. Another group of TH^ARC^ neurons inhibit anorexigenic POMC^ARC^ neurons or activate orexigenic AgRP^ARC^ neurons [206] (Figure 2). Through the anorexigenic and orexigenic neurons, the ARC controls appetite and energy metabolism [197,206,207,208]. A third group of TH^ARC^ neurons releases GHRH, regulating growth and anabolism [195,209]. 

Anorexigenic neurons exert their effects through POMC and CART [195]. Peripheral satiety signals, including insulin and amylin released from the pancreas, glucagon-like peptide-1 (GLP1) released primarily from the gastrointestinal tract, and leptin released from the adipose tissue, reach POMC^ARC^ neurons through the ME [210,211,212,213] (Figure 2). Following activation of these neurons, POMC is cleavaged to α-melanocyte-stimulating hormone (α-MSH) [195]. αMSH is released from POMC^ARC^ neurons that project to the PVN, activating melanocortin-4 receptors (MC4Rs) in the latter [195,214] (Figure 2). Activation of MC4R^PVN^ neurons is involved in the regulation of satiety, energy expenditure, sympathetic activity, blood pressure and growth [214,215,216]. A subset of MC4R^PVN^ neurons projects to the lateral parabrachial nucleus (LPBN), which also receives direct projections from POMC^ARC^ neurons [214] (Figure 2). Glutamatergic^LPBN^ neurons enhance satiety by assigning a positive emotional valence to the satiety state [214]. Interestingly, the MC4R^PVN^→LPBN circuit is sufficient to decrease the homeostatic drive to consume food when there is caloric insufficiency [214]. In addition, POMC^ARC^ project to MC4R^LHA^ and MC4R^BNST^ neurons; however, these targets are dispensable in appetite control [214].

Orexigenic neurons exert their effects through the co-release of AgRP [195]. Peripheral hunger (ghrelin) or satiety (leptin, insulin, GLP1 and peptide YY (PYY)) signals activate or inhibit AgRP/NPY^ARC^ neurons, respectively [217,218,219,220,221,222]. Activated AgRP/NPY^ARC^ neurons release AgRP, which inhibits MCR4^PVN^, inhibiting the satiety signal [223]. In essence, AgRP acts as an antagonist of MC4R, preventing the anorexigenic effects of αMSH on second-order neurons [214,223]. Other effects of the activation of AgRP^ARC^ neurons include insulin resistance and increased locomotor activity in the absence of food [224]. NPY signaling is responsible for a subset of physiological effects of AgRP neurons, such as rapid feeding and regulation of glucose metabolism [224]. NPY is uniquely required for the long-lasting effects of AgRP neurons on feeding behavior and specifically for sustaining hunger in the interval between food discovery and intake [225]. In addition, AgRP/NPY^ARC^ neurons directly inhibit anorexigenic POMC^ARC^ neurons through the corelease of GABA [211,223] (Figure 2). Projections from ARC neurons regulate energy expenditure; POMC neurons increase, whereas AgRP/NPY neurons decrease metabolic activity [195]. Of note, the anorexigenic and orexigenic neurons of the ARC are the best-characterized groups of hypothalamic glucose-sensing neurons, which respond to alterations in the levels of extracellular glucose by modifying their firing rate; POMC neurons are glucose-excited, whereas NPY/AgRP neurons are glucose-inhibited [226,227,228,229,230,231].

POMC^ARC^ and AgRP^ARC^ neurons are reciprocally connected with other hypothalamic nuclei, including the PVN, periventricular region, LHA, DMN, VMN, SON and posterior hypothalamus (PH) [232]. The ARC interacts with the SCN, constituting the SCN-ARC axis; however, it remains unknown which neurotransmitters/neuropeptides participate in this reciprocal interaction [233,234]. The projections of the ARC to the DMN, PVN and LHA are not present at birth but develop progressively postnatally; those to the DMN develop swiftly and early, whereas those to the PVN develop significantly later [235]. The ability of leptin to activate the DMN, PVN and LHA is age-dependent and correlates with the establishment of ARC projections to each of these nuclei [235]. Hence, besides its neuroendocrine role (through its projections to the ME), the ARC seems to be, at least postnatally, the central node of a network involved in the relay of leptin signals to other parts of the hypothalamus, regulating food intake [235]. 

The ARC interacts reciprocally with extrahypothalamic regions, including the BNST, PAG, LC and NTS [232]. Of note, POMC-expressing neurons are also present in the NTS [232]. Although POMC^NTS^ neurons have very different innervation patterns from POMC^ARC^ neurons, there are reciprocal projections between POMC^NTS^ and POMC^ARC^, indicating that these POMC pathways may interact [232]. The ARC/tuberoinfundibular region receives noradrenergic input from the LC and the A1 noradrenergic cell group in the medulla [236]. The ARC receives both excitatory (α_1_, β) and inhibitory (α_2_) noradrenergic projections from the LC [236]. The LC may regulate the neuroendocrine function of the ARC directly via the activation of noradrenergic receptors in the ARC, as well as indirectly via projections of the LC to the PVN [236]. Activation of α_2_ receptors in the ARC regulates the release of GHRH from the latter, and, thus, the release of GH from the adenohypophysis [236]. On the other hand, stimulation of α_1_ noradrenergic receptors activates TH^ARC^ neurons, regulating the release of prolactin from the adenohypophysis [236] (Figure 2). 

The PT is connected with the 3^rd^ ventricle partly through the processes of infundibular recess- and ME-tanycytes [195,237]. The ME corresponds to the superior part of the infundibular stalk [195]. The internal layer of the ME contains the hypothalamic-neurohypophyseal tract (projections from magnocellular PVN and SON neurons) [195]. The external layer of the ME comprises the tuberoinfundibular pathway (projections from parvocellular PVN and ARC neurons) and the superior capillary network of the HPS (Figure 1 and Figure 2). Hence, the PT represents a neuroendocrine interface [195].

#### 5.1.2. The VMN

The VMN is a highly conserved structure across mammals, involved in the regulation of feeding behavior, body weight, glucose homeostasis, sexual behavior and aggression [238]. Although the majority of VMN neurons are glutamatergic, this nucleus contains a heterogeneous combination of neuronal subtypes, including PACAP^+^, Nitric Oxide Synthase 1 (NOS1)^+^, Brain-Derived Neurotrophic Factor (BDNF)^+^, Estrogen Receptor alpha (ERα)^+^, Leptin Receptor (LepR)^+^ and Steroidogenic Factor-1 (SF1)^+^ neurons [238] (Figure 2). SF1 (encoded by NR5A1) is a transcription factor involved in the development of the adrenal glands, pituitary gonadotrope cells, gonads, and the VMN itself [239]. SF1 is expressed in the developing anterior, central and dorsomedial VMN, whereas neurons in the adult ventrolateral VMN do not express SF1 [240,241]. Mice with lesions in the VMN manifest weight gain due to increased food intake and reduced sympathetic outflow [242]. SF1-knockout mice are unable to develop a VMN, whereas animals with selectively knockout-LepR SF1^+^ neurons show increased body weight and adiposity, which is, nonetheless, less severe than that of the generalized LepR loss from all VMN neurons [243,244]. These mice do not respond to a high-fat diet (HFD) by reducing caloric intake or activating diet-induced thermogenesis, indicating that SF1 neurons are probably more important for the adaptation to the obesogenic environment than for the maintenance of the body weight [244]. Interestingly, in the absence of food, low SF1 neuronal activity allows food-seeking behavior, whereas activation of SF1 neurons shifts behavior toward decreased exploration and food avoidance [245].

The VMN is involved in the counter-regulatory neuroendocrine response (CRR) to hypoglycemia, characterized by increased levels of glucagon, GCs and norepinephrine (NE) and decreased levels of circulating insulin [238]. As most VMN neurons are glutamatergic, selective deletion of Vesicular Glucose Transporter (VGLUT) in SF1^+^ neurons leads to an impaired CRR [246]. The neurocircuits that regulate glucose levels consist of glucose-sensing neurons, which intrinsically sense and respond to the levels of glucose in the brain so that they are maintained at ~30% of systemic levels; these neurons can be either glucose-excited or glucose-inhibited [238]. In addition, in the VMN, there are groups of glucose-responsive neurons that are not intrinsically glucose-sensing, including a subgroup of neurons that are presynaptically excited in response to low levels of extracellular glucose, and two other subpopulations that are presynaptically excited or inhibited by high levels of extracellular glucose [247]. The comprehensive roles of different classes of glucose-sensing/-responsive neurons in the VMN remain unknown [238]. In the dorsomedial VMN, glucose-excited and leptin-sensitive neurons may overlap [238]. PACAP^VMN^ neurons are glucose-inhibited [248]. Activation of these neurons inhibits insulin but does not increase glucagon or affect basal glucose levels; however, activation of PACAP^VMN^ increases glucose levels during a glucose tolerance test [248]. In the VMN, glucose-inhibited neurons depend on NO signaling, which provides inhibition in response to rising glucose levels [249]. Interestingly, stimulation of a subset of NOS1^VMN^ neurons that project to the BNST causes hyperglycemia but no freezing behavior, whereas activation of those that project to the PAG causes hyperglycemia and freezing behavior [250,251]. ERα^VMN^ neurons are exclusively present in the ventrolateral VMN and do not express SF1 [238]. These neurons are intrinsically glucose-sensing; via their connections with the ARC and dorsal raphe nucleus (DR), they can modulate circulating glucose levels [238]. ERα^VMN^ neurons are distinct from NOS1^VMN^ and PACAP^VMN^ [248,250]. MCR3^VMN^ neurons that project to the BNST integrate excitatory inputs originating from various neuronal groups involved in processes associated with glucose homeostasis (such as POMC^ARC^ neurons) as well as the LPBN [252].

The VMN is involved in aggressive and sexual behavior [238,253]. Stimulation of ERα/progesterone^VMN^ neurons increases the rate and intensity of aggressive attacks [254,255]. By contrast, stimulation of SF1^VMN^ neurons induces defensive/avoidance behaviors [245,256]. Interestingly, PACAP^VMN^ neurons form an intra-VMN circuit (PACAP^VMN^ neurons in the central VMN project to PACAP^VMN^ neurons in the ventrolateral VMN) (Figure 2), involved in the circadian regulation of aggressive behavior [257]. Glutamate^VMN^ neurons that project to the PAG drive the biting response during aggressive attacks [258]. In female rats, activation of the VMN drives the female lordosis reflex [259,260,261]. In SF1-knockout female mice, the actions of estrogen and progesterone in driving sexual behavior are blocked, thus affecting fertility and reproductive behavior [262]. ERα^VMN^ (projecting to the PAG), Oxt^VMN^ and NOS1^VMN^ neurons are also involved in female sexual behavior [263,264,265,266]. In male mice, optogenetic stimulation of ERα-expressing neurons in the ventrolateral VMN enhances mounting behavior [254]. Interestingly, this effect is intensity-dependent, with high-intensity stimulation inducing aggressive behavior, whereas low-intensity stimulation induces sociosexual behavior [254]. Interestingly, the VMN circuitries that regulate aggressive and sexual behavior overlap [238].

#### 5.1.3. The DMN

The DMN is involved in neuroendocrine and autonomic homeostasis, regulation of feeding and drinking behavior, and body weight [267,268,269,270,271,272]. Rats with DMN lesion syndrome show hypophagia, hypodipsia and reduced ponderal and linear growth, although body composition remains unaffected [267]. Their growth reduction is not associated with a deficiency in growth hormone (GH), insulin-like growth factor 1 (IGF1), thyroid hormones, or insulin [267]. When these rats are fed with a HFD, they do not become as obese as controls, probably because of the downregulation of NPY [267]. Although these rats utilize food efficiently, they show an attenuated response to the feeding-stimulatory effect of insulin [267]. Moreover, they manifest hyperprolactinemia due to reduced dopaminergic signaling, as well as disordered circadian rhythms of wakefulness, feeding, locomotor activity and serum GC levels, accompanied by an overall reduction of GC levels by ~80–90% [269,273]. 

The DMN promotes wakefulness and inhibits sleep through excitatory projections (mainly expressing glutamate, TRH and hypocretin (HCRT)) to orexinergic and non-orexinergic LHA neurons, as well as GABAergic projections to the ventrolateral preoptic nucleus (VLPO) [271,273] (Figure 2). Loss of HCRT or HCRT^LHA^ neurons almost abolishes the circadian rhythm of REM sleep [273]. The DMN and the subparaventricular zone of the hypothalamus (SPZ) constitute the two intermediate stations through which the SCN regulates sleep and behavioral rhythms [273,274]. The ventral SPZ is essential for sleep and locomotor rhythms, while the dorsal SPZ is critical for body temperature rhythms [273,275]. The DMN may act as a functional continuation of the ventral SPZ [273]. Efferent signals from the SCN may utilize the DMN-ventral SPZ complex to regulate the circadian aspect of sleep, locomotor activity and other behaviors, as well as GC release [273,275]. The DMN-SPZ complex may be part of a circadian network, flexibly integrating circadian time with various physiological processes and behaviors so that they are influenced but not constrained by the circadian clock [273]. Interestingly, both the DMN and VMN (the latter projecting to the SPZ) express LepRs involved in the regulation of feeding and body weight [276]. 

The PVN is the major target of the DMN [271]. The DMN may be part of a PVN-centered circuitry regulating neuroendocrine and autonomic homeostasis, such as the cardiovascular response to stress [269,272]. In addition, the DMN projects to the dorsal region of the periventricular zone and the SCN, while it is reciprocally connected with the ARC, VMN and LHA [268,271]. The DMN projects to the area surrounding the organum vasculosum of the lamina terminalis (OVLT) and the subfornical organ (SFO) [271]. Nonetheless, only a few DMN neurons project to the vascular organ proper [271]. The DMN densely projects to extrahypothalamic regions involved in the regulation of anxiety, sexual behavior and visceromotor functions [271,272]. Nonetheless, it is unknown whether the role of these efferent fibers is the circadian modulation of the associated functions [273]. The descending projections of the VMN follow primarily two pathways that converge at various levels: (i) a dorsal pathway in the periventricular system of the midbrain spans through and mainly innervates the periaqueductal gray (PAG) and pontine gray nuclei, while (ii) a ventral pathway extends through the ventromedial brainstem [271] (Figure 2).

### 5.2. The SCN

#### 5.2.1. Cellular Time-keeping and Biological Rhythms

In mammals, biological rhythms are regulated by a group of hierarchically organized oscillators [277]. Although there is a common system of molecular mechanisms (molecular clock) that drives biological rhythms across all oscillators in mammalian organisms, this system flexibly adapts to the specific molecular or tissue context [277]. The molecular clock is cell autonomous, organized around an autoregulatory transcriptional/translational network of negative feedback loops (transcriptional-translational feedback loops (TTFLs)) [278]. The Circadian Locomoter Output Cycles Protein Kaput (CLOCK) and Brain and Muscle ARNT-like protein-1 (BMAL1) are transcription factors that act as the hubs of this network, driving rhythmic gene expression [277,278]. The cycle lasts ~24 h [278]. At the beginning of the cycle (circadian time (CT) = 0), CLOCK and BMAL1 form heterodimers (CLOCK:BMAL1) that rhythmically upregulate the Period (PER1 and PER2) and Cryptochrome (CRY1 and CRY2) genes, through the binding of the CLOCK:BMAL1 complex to enhancer-box (E-box) regulatory sequences [278]. PER and CRY genes act as negative regulators (transcriptional repressors); at the middle of the cycle (CT = 12), the products of PER and CRY accumulate and dimerize, forming a complex that translocates into the nucleus to interact with CLOCK:BMAL1, repressing their own transcription [278]. During the last half of the cycle (CT = 12 to CT = 0), PER and CRY are downregulated, and the existing PER-CRY complexes are degraded, allowing the re-initiation of the cycle at CT = 0 (24 h after the initiation of the previous cycle) [278]. The CLOCK-BMAL1/PER-CRY loop is stabilized by accessory feedback loops, such as the REV-ERB/RORα loop [278,279]. REV-ERB and RORα are nuclear receptors that act as transcriptional targets of the CLOCK:BMAL1 via Rev response elements (RREs) [278,279].

The SCN acts as the central pacemaker of the mammalian clock [277]. Each SCN neuron contains a cell-autonomous circadian oscillator [277]. Individual SCN neurons can maintain cell-autonomous circadian cycles of spontaneous firing rate (SFR), intracellular Ca^++^ concentration and gene expression, controlled by their TFFLs, which are, nonetheless, poorly organized [280,281]. On the other hand, when SCN neurons are interconnected within a network, cellular interactions increase the stability, coherence and amplitude of TFFLs, leading to precisely synchronized rhythms of gene expression and neuronal activity that can be maintained indefinitely [277,281,282,283]. In SCN neurons, TTFLs couple with electrical activity through alterations in the Na^+^ and K^+^ conductance of the plasma membrane and in the levels of intracellular Ca^++^, distinguishing daytime activity from nocturnal quiescence [281,284]. Changes in the levels of intracellular Ca^++^ couple the electrical activity to TTFLs via Ca^++^/cAMP Response Elements (CREs) in PER genes; thus, circadian inputs become inputs to the TTFLs, increasing the stability and precision of the latter [281,284]. During daylight, there is strong electrical-transcriptional/metabolic coupling, whereas, during the night, there is electrical and metabolic quiescence as well as astrocytic activation [281]. 

The entrainment of mammals to the light-dark cycle is effectuated by the increased firing of the SCN, mediated by the retinohypothalamic tract (RHT) [281]. The output metric of the SCN is the SFR, oscillating in a spectrum of frequencies (>10 and <1 Hz) [283,285]. The SFR encodes solar time; changes in the SFR drive circadian rhythms, with electrical and metabolic activity being higher during the circadian day [281]. 

#### 5.2.2. Structural and Functional Organization of the SCN

The SCN is located against the 3^rd^ ventricle above the optic chiasm (OC) [286] (Figure 2). The SCN is connected with hypothalamic and brainstem centers that control feeding behavior, sleep and arousal as well as the neuroendocrine and autonomic response, via efferent projections that combine in an SFR-dependent fashion the synaptic release of GABA (from all SCN neurons) with the paracrine release of neuropeptides (from specific groups of SCN neurons) [281] (Figure 2). 

According to the linear model (input→oscillator→output), the SCN is divided into a core and a shell subregion; the former receives input from the retina through the RHT, and is weakly rhythmic, whereas the latter shows predominant oscillations and sends extensive efferent projections [281]. In the core, the main neuropeptides are VIP and Gastrin-Releasing Peptide (GRP), whereas, in the shell, the main neuropeptide is AVP [281,287,288]. Light activates the intrinsically photosensitive retinal ganglion cells (ipRGCs) of the RHT, which express the photopigment melanopsin and release glutamate and PACAP [281]. Glutamate^RHT^ activates glutamate receptors in SCN core neurons [289]. Activated core neurons propagate the action potentials to shell neurons via GABA and neuropeptides, including VIP and GRP [284,290]. VIP^SCN^ core neurons activate AVP^SCN^ shell neurons, which are responsible for the synaptic and molecular output [290] (Figure 2). The depolarizing effects of the RHT depend on the phase of the day; when the light is delivered during the daytime (when the SFR is already high), there is no sustained effect on the clock [281]. On the other hand, brief bouts of light delivered during early or late circadian night increase firing, delaying the fall or enhancing the rise of the SFR; thus, the peak in the SFR is always at dawn or dusk illumination [281]. The function of intra-SCN circuits depends on VIP released from core neurons, which activates VPAC2 on shell neurons [281,290]. Deletion of genes encoding VIP or VPAC2 desynchronizes the network, whereas activation of VIP neurons re-establishes and re-directs the wave of circadian gene expression [291,292]. The combined effects of glutamate (released from the RHT), VIP (released from core neurons) and GABA (released from all neurons) maintain the properties of the SCN circuit; of note, synchrony depends on the balance between VIP and GABA, with VIP promoting whereas GABA opposing synchrony [281,293,294]. 

According to the modular model, the SCN shell consists of modules of SCN neurons that interact reciprocally [290]. In module 1, AVP^+^ neurons coexpress VPAC2 receptors (these neurons are distributed ventrodorsally and located dorsally to the core VIP^+^ neurons) [290]. In module 2, PACAP^+^ neurons coexpress AVPR and NPYR (distributed throughout the SCN) [290]. In module 3, neurons coexpress Proprotein Convertase Subtilisin/Kexin type 1 inhibitor (PCSK1N), GABAA receptor subunit alpha1 (GABA_A1_) and PAC1 (distributed throughout the SCN, similarly to neurons of module 2) [290]. According to this model, the most significant SCN neuropeptides are VIP, AVP, PACAP and PCSK1N, produced by core neurons and neurons in modules 1, 2 and 3 of the shell, respectively (Figure 2) [290]. 

VIP has traditionally been considered an input mediator and network coordinator [281,290]. Besides the intrinsic rhythmicity of VIP^SCN^ neurons, these neurons project (directly or indirectly) to targets, including the paraventricular thalamus, SPZ, PVN, medial preoptic nucleus (MPO), DMN, VMN and ARC (Figure 2) [295]. Hence, VIP^SCN^ neurons may provide information about circadian timing to neural circuits that contribute to the control of the neuroendocrine and autonomic response [295]. Through these circuits, the SCN may control the daily rhythms of core physiological processes, such as the heart rate and GC release [295]. Indeed, target neurons demonstrate circadian variations during the day, with their nadir coinciding with the peak in endogenous VIP^SCN^ neuronal activity [295]. Thus, VIP^SCN^ neurons may inhibit the activity of specific groups of target neurons via synchronized waves of GABAergic (rather than VIPergic) activity [295]. Interestingly, the timing of peak firing is more variable in target neurons than in VIP^SCN^ neurons [295]. This phenomenon may be explained by the existence of local clocks in target neurons [295].

### 5.3. The PVN

The PVN is one of the most complex and cellularly heterogeneous CNS nuclei [296]. Despite the remarkably high evolutionary conservation of the hypothalamus in vertebrates [173], the cellular organization in the PVN differs substantially between rats and mice [296,297,298,299,300,301,302,303,304,305,306]. The structural differences in two such closely related species may have mechanistic implications for brain evolution as well as for the interpretation of functional and behavioral differences between species [296]. Overall, the PVN comprises three main types of neurons: (a) parvocellular neuroendocrine cells, (b) magnocellular neuroendocrine cells, and (c) long-projecting neurons [296]. These subpopulations broadly correspond to the three primary subdivisions of the PVN: parvocellular, magnocellular and descending [296]. In mice, the rostral 2/3 of the PVN contains the majority of parvocellular and magnocellular neurons, whereas the caudal 1/3 contains most of the descending preautonomic neurons [296] (Figure 2).

Parvocellular neurons synthesize hormone release-stimulating or -inhibiting neuropeptides, such as CRH, TRH and somatostatin [296]. In rats, a few parvocellular neurons also synthesize and release GHRH and dopamine [302]. In both rats and mice, most CRH neurons are distributed in the dorsal zone of the medial parvocellular part [296]. In mice, somatostatin-expressing neurons are distributed in the periventricular zone along the wall of the 3^rd^ ventricle [296]. Parvocellular neurons project to the ME, releasing their neuropeptide output into the fenestrated capillaries of the HPS, regulating the synthesis and release of ACTH, thyroid-stimulating hormone (TSH) and GH from the adenohypophysis, thus constituting the subaxes of the hypothalamic-hypophyseal (pituitary) axis: (i) the hypothalamic-pituitary-adrenal (HPA) subaxis, (ii) the hypothalamic-pituitary-thyroid (HPT) subaxis and (iii) the hypothalamic-pituitary-growth hormone subaxis, respectively [296]. ACTH stimulates the release of GCs from adrenal glands, TRH stimulates the release of thyroid hormones from the thyroid gland, and GH stimulates the release of IGF1 from peripheral tissues [156]. In rats and mice, most CRH^PVN^ and somatostatin^PVN^ neurons are neuroendocrine, whereas TRH^PVN^ neurons comprise equal numbers of neuroendocrine and non-neuroendocrine neurons [296]. The majority of TRH neurons are distributed in the dorsal zone of the medial parvocellular part, adjacent to the periventricular portion of the PVN [296,302].

Magnocellular neurons project primarily to the neurohypophysis, releasing Oxt and AVP into the systemic circulation [296]. Oxt- and AVP-expressing neurons are distributed in distinct parts of the magnocellular subdivision of the PVN [296]. The anterior and medial magnocellular parts contain almost exclusively Oxt neurons, whereas the posterior magnocellular part contains both Oxt and AVP neurons [296]. In both mice and rats, the lateral zone of the posterior part mainly contains AVP neurons [296,297]. In mice, although some parvocellular CRH neurons may intermingle with magnocellular neurons (at least in the borders of the two zones), only a few CRH neurons colocalize with Oxt or AVP neurons [296]. By contrast, in rats, ~40% of Oxt neurons in the anterior magnocellular part coexpress CRH [306]. In mice and rats, CRH is generally not coexpressed with AVP [296,306]. However, adrenalectomy increases the coexpression of CRH and AVP in parvocellular neurons [296,305]. In the human PVN, parvocellular neurons express CRH and AVP, whereas magnocellular neurons express AVP [307].

The descending division of the PVN consists of preautonomic neurons that project to the lower brainstem and spinal cord (Figure 2) [296]. It has been proposed that this division coordinates sympathetic and parasympathetic activity, and integrates the autonomic with endocrine activity [296,297,300,301,302,303]. Three descending preautonomic neuronal subpopulations project to (a) the intermediolateral column of the spinal cord (IML), (b) the dorsal vagal complex (DVC) (constituted by the DMV, NTS and area postrema (AP)), and (c) the central gray of the spinal cord (CGS) (which surrounds the central canal at the cervical and upper thoracic levels) [296]. In addition, the descending division of the PVN comprises neurons that project to midbrain and brainstem regions, such as the PAG, rostroventral medulla (RVM) and parabrachial nucleus (PBN) [308,309,310,311]. Of note, the area around the CGS contains fibers expressing neurophysin 1, Oxt and AVP, which probably originate from PVN neurons [296]. Remarkably, in mice, descending neurons (especially IML-projecting) have abundant dendrites that extend to enclose the PVN from the outside at levels of the magnocellular and parvocellular subdivision, resulting in the complete ensheathment of these neuroendocrine PVN areas by the complex dendritic branches of pre-autonomic descending neurons in a nest-like manner [296]. It has been proposed that this nest-like conformation may constitute an interface of neuroendocrine-autonomic coordination [296]. 

Parvocellular CRH^PVN^ neurons project densely to the LC; CRH increases the activity of noradrenergic LC neurons, thus enhancing the sympathoexcitatory effect of the latter [236]. In the opposite direction, the LC sends a dense excitatory noradrenergic projection to the PVN [236]. The PVN along with the pontine noradrenergic nuclei (LC/A5-A7) and RVLM is part of an autonomic network that regulates the activity of preganglionic neurons [312,313]. Of note, the LC/A5-A7 complex (a) acts as sympathetic premotor nuclei through the stimulation of α_1_-adrenoreceptors in preganglionic sympathetic neurons in the IML, (b) acts as parasympathetic premotor nuclei, projecting to and stimulating α_2_-adrenoreceptors in preganglionic parasympathetic nuclei in the brainstem, and (c) modulates the activity of other sympathetic premotor nuclei, such as the PVN and RVLM [236].

The PVN is a central node of the appetite-suppressing axis [214]. MC4R^PVN^ neurons project to glutamatergic neurons of the LPBN, enhancing satiety [214]. In addition, MC4R^PVN^ project to the ME, NTS, DMV, and more sparsely to the ventrolateral periaqueductal gray (vlPAG), LC, RVLM and spinal cord [214] (Figure 2). 

According to the linear model, the PVN receives inputs from other CNS regions; these inputs linearly relate to the neuroendocrine and/or autonomic PVN output [314]. However, within and around PVN, there is a circuitry of glutamatergic and GABAergic interneurons, which modulate and integrate the neuronal input with the neuroendocrine/autonomic output [314]. Glutamatergic interneurons are located within the PVN, whereas GABAergic interneurons are situated mainly in a halo zone around the nucleus, regulating the excitability of PVN neurons [314]. However, GABAergic interneurons are occasionally present within the PVN [314] (Figure 2). Interestingly, CRH released from parvocellular CRH neurons activates a distinct population of CRH receptor 1 (CRHR1)^+^ neurons in the PVN (Figure 2) [315]. These neurons send recurrent GABAergic projections to CRH^PVN^ neurons to decrease the excitability of the latter, thus modulating the HPA axis response to stress [315]. It has been proposed that CRHR1^PVN^ neurons may be involved in neuroendocrine-autonomic coordination [315,316]. 

Νeuropeptides and signaling molecules, including angiotensin and HCRT released from non-synaptic sites of PVN neurons and/or from glial cells, as well as originating from other hypothalamic or extrahypothalamic regions, may contribute to the shaping and integration of PVN outputs [314]. For example, neurons originating from the SFO provide excitatory input to IML-projecting PVN neurons, with angiotensin being one of the major neuropeptides of this pathway [314] (Figure 2). HCRT^LHA^ neurons project to the PVN, influencing descending preautonomic neurons, and parvocellular and magnocellular neurons [314]. Hence, angiotensin and HCRT may regulate autonomic activity and/or the release of stress hormones [314].

#### The Entrainment of CRH^PVN^ Neurons by the SCN

The daily rhythm in the levels of circulating GCs is dependent on the coordinated expression of clock genes associated with rhythms of neuronal activity in both CRH^PVN^ and VIP^SCN^ neurons [317]. In vivo, CRH^PVN^ neurons show sustained circadian rhythms of gene expression, depending on the phase of the light cycle, with BMAL1 regulating the cell-autonomous circadian regulation of the excitability in CRH^PVN^ neurons [317]. Loss of BMAL1 in CRH^PVN^ neurons results in arrhythmic Ca^++^ activity in these neurons and decreases the amplitude and reliability of the release of GCs by the adrenal glands [317]. However, the expression of BMAL1 in CRH^PVN^ is not sufficient for rhythmic Ca^++^ activity and GC release [317]. SCN lesions or BMAL1 deletion in all SCN neurons abolish the circadian GC rhythm, which is dependent on the firing of SCN**^PVN^** neurons, the latter entraining the PVN to its intrinsic daily rhythms [317,318,319]. Activation of VIP^SCN^ neurons can acutely inhibit CRH^PVN^ neurons and the release of GCs by the adrenal glands [317]. Hence, SCN^VIP^ neurons exert both a circadian and an acute effect on PVN^CRH^ neurons [317]. Double-labeled immunohistochemistry showed that VIP^SCN^ neurons end near the VPAC2^+^ CRH^PVN^ neurons (Figure 2); the former release VIP, which potentially reaches (through volume transmission) and activates VPAC2 in the latter [317]. Alternatively, PVN**^CRH^** may be the target of GABA released from VIP**^SCN^** or other SCN neurons [295,317]. Overall, the neural circuit that regulates the peripheral circadian release of GCs by the adrenal glands includes entraining and inhibitory signals from VIP^SCN^ neurons onto intrinsically circadian CRH^PVN^ neurons [317].

### 5.4. The Hypothalamus as a Regulator of Adult Neurogenesis

POMC^ARC^ neurons send long-range projections to the anterior ventral ventricular-subventricular zone (AV V-SVZ) [320]. In adult mice, satiety and hunger signals regulate the proliferation of NKx2.1^+^ NPCs in the AV V-SVZ by modulating the activity of POMC^ARC^ neurons [320]. Fasting inhibits POMC^ARC^ neurons, whereas feeding activates them [320]. Fasting decreases the proliferation of NKx2.1^+^ NPCs in the AV V-SVZ, whereas refeeding recovers NPC proliferation at basal levels; the number of IPCs and neuroblasts remains unaffected [320]. The homeodomain transcription factor NK2 homeobox 1 (Nkx2.1) controls the differentiation of telencephalic GABAergic interneurons and oligodendrocytes and regulates astrogliogenesis by binding the promoter of GFAP [321]. In addition, NKX2.1 is involved in the early morphogenesis of the developing hypothalamus and the establishment of the early identity of melanocortinergic neurons by activating the expression of POMC [322].

The supramammillary nucleus (SuM), lying immediately dorsally to the mammillary, is considered a major node of a sleep−wakefulness regulatory system [323,324]. DG-projecting SuM neurons respond to environmental novelty by increasing their activity and firing frequency [324]. In mice, chronic SuM activation increases the production of DG NPCs and their maturation to young adult-born neurons, whereas chronic SuM inhibition has the opposite effect [323]. SuM neurons corelease glutamate and GABA, and densely project to the DG, enhancing the output of granule cells [324,325]. Interestingly, SuM neurons initially provide glutamatergic input to NPCs, then GABAergic input to immature neurons, and finally combined glutamatergic/GABAergic to adult-born neurons [324,325].

PVN neurons project directly to the CA3 region of the hippocampus [326]. In the adult DG, Oxt promotes neurogenesis [326]. However, Oxt receptors (OXTRs) are not expressed in SGZ NPCs or mature DG granule cells [326]. On the other hand, hippocampal NPCs express functional CRH receptors (CRHRs); this characteristic is highly conserved in mammals [327]. Genetic deficiency of CRH synthesis negatively affects hippocampal neurogenesis and the responsiveness of NPCs to environmental stimuli and is associated with impaired spatial memory [327]. The local disruption of the CRH/CRHR system reduces neurogenesis, whereas exposure of adult NPCs to CRH promotes neurogenesis by suppressing BMP4 [327]. In an experimental Alzheimer’s disease (AD) model, stimulation of MC4Rs enhanced DG neurogenesis through activation of the canonical Wnt-3A/β-catenin and Shh signaling pathways; this effect was associated with the rescue of cognitive decline [328,329]. In another study, orexinergic stimulation enhanced DG neurogenesis through activation of orexin receptors type 1 (OX1Rs) and subsequent activation of ERK1/2 [330,331]. 

## 6. The CVOs

The CVOs are specialized midline or near-midline structures (located around the 3^rd^ or 4^th^ ventricle) with a unique cytoarchitecture comprising neurons, glial cells, blood vessels and leptomeningeal components [332,333]. The capillaries at the CVOs are fenestrated, and perivascular spaces are large; thus, the BBB is incomplete at the CVOs [332,334,335,336]. The fenestration of the endothelial cells of capillaries results from the downregulation of tight junction and transport proteins [332]. At the CVOs, modified ependymal cells, including tanycytes, line the ventricular wall [332,335]. The cell bodies of CVO tanycytes are interconnected with tight junctions, acting as a diffusion barrier, thus shifting the BBB function from the capillary to the ventricular wall [332,337].

The CVOs can be broadly classified into sensory and secretory [332]. The SFO, OLVT and AP are sensory, whereas the ME, neurohypophysis and pineal gland are secretory CVOs [332]. Of note, the subcommissural organ (SCO) (a poorly characterized CVO) uniquely lacks fenestrated capillaries and is considered both sensory and secretory [332]. Sensory CVOs mainly contain neuronal somata, which come into contact with peripherally circulating molecules, thereby transducing chemical signals into electrical [332]. After that, the projections of sensory CVO neurons deliver the transduced information to various CNS nuclei [332]. On the other hand, secretory CVOs primarily consist of axons and nerve terminals, which release bioactive peptides into the systemic circulation [332]. 

The CVOs are characterized by restrictive, size-dependent vascular permeability rather than a complete lack of BBB [334]. Fenestrated capillaries allow low molecular weight molecules (from the systemic circulation) to diffuse passively into the CVOs [334]. By contrast, high molecular weight molecules, such as several protein hormones and cytokines, are not able to freely enter the CVOs but instead utilize transcytosis systems at the BBB [336,338]. These systems are regulated by CNS-specific genetic programs, which, at baseline, inhibit transcytosis so that a functional barrier is maintained [336,338]. Transcytosis is most prominent in the MBH [338]. Interestingly, plasma leptin (a high molecular weight biomolecule) enters the CNS through transcytosis, exclusively via the activation of LepRs in ME tanycytes [339] (Figure 2, Inset 2). 

Microglial cells and macrophages are over-represented in the CVOs compared to other brain regions [340]. Interestingly, CVO microglia are amoeboid in the basal state, in contrast to other brain regions in which resting microglia (M0) is ramified [341]. Amoeboid microglia represent a phenotype at the end of the spectrum of microglial activation states [341]. Consistently, microglia in the CVOs express higher levels of M1 (activated microglia) and M2 (alternatively activated microglia) marker proteins than microglia in other brain regions [340]. The role of continuous microglial activation in the CVOs remains unclear; phagocytosis of blood-borne molecules and regulation of angiogenesis and neurogenesis are among the proposed roles [340]. Interestingly, in adult mice, the expression of Toll-like receptors 4 and 2 (TLR4 and TLR2) is markedly higher in the CVOs than in other brain regions [340]. In the CVOs, TLR4 is highly expressed in astrocytes and tanycytes [340].

### 6.1. The SFO

The SFO is a small ovoid structure situated in the midline anterior dorsal wall of the 3^rd^ ventricle, dorsally to the anterior commissure, at the junction of the foramina of Monro, and in close vicinity to the choroid plexus [332,342] (Figure 1b,d and Figure 2). In the human brain, the SFO is minuscule, observable in the wall of the 3^rd^ ventricle as a slight eminence just below the tela choroidea of the choroid plexus [335,342]. 

Nerve fibers from the SFO travel through the MPO to the preoptic region, SON, SCN and OVLT [342]. The SFO projects densely to the parvocellular and magnocellular subdivision of the PVN [342]. In addition, the SFO projects to the LHA, ARC/ME, and dorsal perifornical area [342]. The SFO projects to extrahypothalamic regions, including the midline thalamic nuclei, zona incerta, BNST, raphe nuclei, CeA, and the infralimbic prefrontal cortex [332,342]. The SFO mainly receives input from brainstem centers, including the LC, raphe nuclei, LPBN, NTS, ventrolateral medulla (VLM), and laterodorsal tegmental nucleus [342]. The SFO receives input from hypothalamic nuclei, including the MPO, ARC, DMN and PVN, as well as from extrahypothalamic sites, including the thalamic nucleus reuniens, BNST and PAG [332,342].

Interestingly, the SFO is the CNS region with the highest density of a variety of types of neuropeptide receptors [335]. Nonetheless, it remains unknown whether distinct SFO neuronal subpopulations show functional specialization [335]. One well-characterized subpopulation expresses angiotensin II type-1 receptors (AT1Rs) and is involved in drinking, pressor response and salt appetite through projections to the MPO, SON, PVN and BNST [335]. Another major subpopulation expresses nNOs and is involved in the initiation of thirst [335]. 

### 6.2. The OVLT

The OVLT is situated in the midline, in the anterior wall of the 3^rd^ ventricle, immediately dorsally to the optic chiasm (OC), extending towards the anterior commissure [342] (Figure 2). Rostrally, the OVLT is attached to the pial surface of the CSF-filled pre-chiasmatic cistern, with the intrapial capillary plexus invaginating into the body of the OVLT [342]. Caudally, the base of the OVLT protrudes into the optic recess (OR) of the 3^rd^ ventricle [342]. Roughly, the OLVT can be divided into a rostromedial vascular region, a dorsal cap and a lateral/posterior region that projects to CNS nuclei [342]. 

The OVLT projects directly and indirectly (via the MPO) to magnocellular neurons of the PVN and SON and parvocellular PVN neurons [342] (Figure 2). The OVLT projects to the lateral preoptic area, LHA, parastrial nucleus, BNST, LPBN, cingulate cortex and hippocampus [342]. The OVLT sends polysynaptic efferent projections to peripheral organs and sympathetic ganglia through the PVN, SCN and, possibly, through other relay stations not yet identified [342]. The OVLT receives input from the SFO, MPO, PVN, VMN, ARC, posterior hypothalamic area (PHA), and midbrain/brainstem regions, including the PAG and LC [342]. In addition, there are intra-OVLT connections between OVLT neurons [332].

In primates, the ventricular wall at the OVLT is lined by modified ependymal cells, which are flattened, elongated and non-ciliated without protrusions to the ventricles [342]. Ependymal cells are in contact with neuron-like supra-ependymal cells, the processes of which cross the surface of the OVLT [342]. 

### 6.3. The AP

The AP lies on the wall of the 4^th^ ventricle, on the dorsal surface of the medulla oblongata near the opening of the central canal, adjacent to the NTS [342]. Rostrally, the AP connects to the choroid plexus [342]. Unlike the other CVOs, the AP receives direct input from sensory nerves, including the vagal and glossopharyngeal [332,342]. The AP receives some input from the CNS, mainly the PVN, periventricular area, anterior part of the DMN, and perifornical region [342]. The major efferent branch from the AP proceeds to the A1 noradrenergic group of the caudal ventrolateral medulla, with some fibers terminating at the nucleus ambiguus [342]. This branch reaches the LPBN (the major terminal), while other fibers end at the PAG [342]. The AP is a part of the DVC, which integrates afferent signals from the gut and viscera [342]. The AP is separated from the NTS by the funiculus separans, a region enriched with glial cells [342]. The AP projects to the NTS, whereas only a few AP neurons end at the DMV [342]. The AP projects to the SCN through a polysynaptic pathway [342].

### 6.4. CVOs and NPCs

In adult rats, fenestrated capillaries in the ventromedial core of the SFO are mainly in contact with tanycytic processes that express high concentrations of vimentin and low concentrations of GFAP [335]. On the other hand, unfenestrated capillaries in the dorsolateral core are primarily in contact with GFAP^+^/vimentin^-^ astrocytic processes [335]. A similar organization has been observed in the ME and OVLT [335] (Figure 2). 

In adult mice, the CVOs contain radial ependymoglial tanycyte-like cells that can constitutively proliferate and differentiate into neurons and glia and can self-organize into neurospheres [343]. These cells express NPC markers such as nestin, vimentin, GFAP and SOX2, while a subpopulation of SOX2^+^ cells coexpress Ki67 [343]. SOX2^+^/Ki67^+^ cells may represent quiescent NPCs with the potential to amplify and differentiate under specific conditions [343]. Of note, proliferating NPCs in the SFO, OVLT and ME can generate astrocytes, whereas AP NPCs can give rise to both neurons and astrocytes [332]. 

In adult mice, sensory CVOs contain either EGFP^+^ tanycyte-like ependymal cells or EGFP^+^ astrocyte-like NPCs, which can give rise to neurons and glial cells within the CVOs themselves as well as in the neighboring regions [344]. Of note, tanycyte-like ependymal cells have similar morphology to ME tanycytes [344]. Interestingly, NPCs originating from the SFO, OVLT and AP can migrate to the ventromedial hippocampal commissure, MPO and NTS/hypoglossal nerve, respectively [344]. Hence, sensory CVO NPCs may supply the neighboring CNS parenchyma (or even the PNS) with newly generated neurons and glial cells [344]. In addition, through their secretome, CVO NPCs may contribute to the reconstruction of the CVO parenchyma and blood vessels through the release of VEGFA and VEGFC [344].

In the adult mouse SFO, OVLT and central canal, tanycyte-like ependymal cells express nestin, SOX2 and GFAP [345]. In the central canal, tanycyte-like ependymal cells can form neurospheres that can self-renew and differentiate mainly into astrocytes and oligodendrocytes [345]. Of note, CNS injury increases the proliferation of tanycyte-like ependymal cells [345]. It is plausible that NPCs around the ventricles and central canal (i.e., the peri- and para-ventricular portions of the neurogenic niches) act as an endogenous repair system, sensing mechanical and chemical information from the CSF, thus regulating neurogenesis and gliogenesis accordingly [345]. As the CVOs are close to the CSF and contain fenestrated capillaries, these structures may sense homeostatic aberrations originating from the nervous system and the periphery of the organism [343].

In the adult human brain, the ME, neurohypophysis, pineal gland and AP contain cells expressing NPC markers such as nestin, GFAP, vimentin, oligodendrocyte transcription factor 2 (OLIG2) and PSA-NCAM, while a proportion of these cells coexpress Ki67 [346]. In the postmortem brain of patients with a history of ischemic stroke, there is an increased number of Ki67^+^ and OLIG2^+^ CVO cells close to the ischemic lesions [346].

## 7. The Hypothalamic Neurogenic Niche

### 7.1. Animal Models

The existing knowledge on the organization of the adult hypothalamic neurogenic niche, including the identity, lineage relationships and localization of different types of hypothalamic NPCs and their progeny, as well as the functional role of hypothalamic neurogenesis, is still elementary compared to that about the SVZ and SGZ [16]. Most evidence comes from studies in animal models focusing on the MBH, probably due to the fundamental role of this region in feeding and energy metabolism [8]. There is evidence that the postnatal/adult mammalian hypothalamus contains a neurogenic niche in which NPCs can proliferate and differentiate into neurons, astrocytes and oligodendrocytes, and integrate into the existing neural circuits [8,16]. 

In adult rodents, there are at least three hypothalamic neurogenic subniches: (a) a tanycytic, (b) a parenchymal, and (c) an IGF1-responsive [16]. Tanycytes have traditionally been classified into four subtypes (α_1_, α_2_, β_1_ and β_2_) depending on their location, morphology, molecular and functional profile [16,19,347,348]. β tanycytes generate neurons that populate the ME and MBH, whereas α tanycytes mainly give rise to parenchymal astrocytes and a few neurons, as well as to β tanycytes [347,348,349,350] (Figure 2, Inset 2). Parenchymal NPCs are scattered within the MBH parenchyma surrounding the 3^rd^ ventricle [16]. These cells comprise three subpopulations: SOX2^+^/Neuron-Glial antigen 2 (NG2)^+^, SOX2^+^/NG2^−^ and SOX2^−^/NG2^+^ cells [16]. SOX2^+^ cells show broader multipotency than NG2^+^ cells [16]. SOX2^+^ NPCs have the potential to generate neurons, astrocytes and oligodendrocytes, whereas NG2^+^ NPCs can generate primarily oligodendrocytes and a few neurons [16,348,349]. Nonetheless, the neurogenic potential of NG2^+^ NPCs remains controversial [351]. Although the terms “NG2^+^ cells” and “OPCs” are used interchangeably, NG2^+^ cells represent a fourth CNS glial subpopulation besides astrocytes, oligodendrocytes and microglia [351]. Of note, NG2^+^ glia represent the largest proliferative cellular subpopulation homogeneously distributed in the adult CNS [351]. In the mid-3^rd^ ventricle of adult rats, there is a region containing subependymal astrocytes and GFAP^+^ tanycytes that proliferate in response to the infusion of IGF1 [352]. Subependymal astrocytes have one or two cilia and a long process inserted between ependymal cells, reaching the CSF [352]. In the same region, labyrinths of basement membranes emanate from capillaries similarly to the SVZ [16,352]. In this region, the wall of the 3^rd^ ventricle consists of three cellular layers: a layer of ependymal cells, a layer of subependymal astrocytes and a deeper layer of tanycytes; of note, this region is absent in mice [16,352]. Ref. [353] recently showed that, in the mouse postnatal hypothalamus, there is a subtype of RGLCs (distinct from tanycytes) in the floor and along the dorsal wall of the 3^rd^ ventricle, interspersed among α and β tanycytes [353]. These cells highly express Iroquois-Class Homeodomain Protein 3 and 5 (IRX3 and IRX5) and behave like NPCs, being able to differentiate into tanycytes and neurons [353]. In the adult hypothalamus, IRX3^+^/IRX5^+^ RGLCs are found in the floor of the 3^rd^ ventricle interspersed with β tanycytes [353] (Figure 2, Inset 2). IRX3^+^/IRX5^+^ RGLCs express SOX2 and quiescence markers but show weak expression of GFAP [353]. Of note, the downregulation of IRX3/IRX5 in IRX3^+^/IRX5^+^ RGLCs results in an increased number of leptin-sensing neurons in the ARC, enhancing the hypothalamic response to leptin [353]. 

In adult sheep, SOX2^+^, GFAP^+^ and vimentin^+^ NPCs in the ME-ARC complex are predominantly distributed in the ependymal and subependymal layers lining the 3^rd^ ventricle [140]. In the ME, most NPCs express high levels of SOX2 [140]. The majority of SOX2^+^ NPCs are located in the ependymal and subependymal layers, whereas a low proportion of them is organized in small chain-like clusters in the hypothalamic parenchyma [140]. The radial processes of almost all NPCs express vimentin; most of these processes coexpress GFAP [140]. In the ME-ARC, the long processes of NPCs penetrate the parenchyma; this conformation is reminiscent of that of tanycytes [140]. GFAP^+^/vimentin^−^ cells are more numerous in the ME than in the ARC [140]. SOX2^+^/nestin^+^ cells (with typical tanycytic morphology) line the wall of the 3^rd^ ventricle at the level of the ventral and dorsal ARC and the ME [140]. 

The adult rodent and sheep SZV/OB and SGZ show similar DCX expression patterns [140]. By contrast, the adult rodent and sheep hypothalamus have different DCX expression patterns [140]. In the adult rodent hypothalamus, DCX expression ranges from moderate to high in the ME (mainly in fibers) but is lower in the ARC [140]. By contrast, in the adult sheep hypothalamus, the expression of DCX is remarkably high in the ME-ARC but low in the VMN and the DMN [140]. In sheep, DCX^+^ cells show heterogeneous morphologies [140]. In the ME-ARC, most of these cells are small, round-shaped, intensely labeled neuroblasts, with some of them forming clusters of densely packaged cells [140]. At the ARC-VMN border, DCX^+^ cells are more fusiform; some of these cells are unipolar with a long and densely labeled process [140]. In the VMN, DCX^+^ cells are fusiform unipolar or bipolar with thick, long processes [140]. In the ME, DCX^+^ cells coexpress PSA-NCAM [140]. By contrast, in the ARC, only some DCX^+^ cells coexpress PSA-NCAM [140]. Most medium- to large-sized DCX^+^ cells are unipolar or bipolar and coexpress HuC/D [140]. Large DCX^+^ cells with thick processes show strong DCX staining, whereas cells with strong HuC/D staining show weaker DCX staining, indicating different maturational stages [140]. Some DCX^+^ cells coexpress ERα; a subgroup of DCX^+^/ERα^+^ cells also coexpress NPY [140]. In addition, in sheep, there are cells coexpressing nestin/DCX or SOX2/DCX [140]. Of note, in mice and sheep, the expression pattern of PCNA is similar in the ME and ARC [140]. However, in mice, there are fewer PCNA^+^ cells, indicating a lower level of ongoing neurogenesis [140].

#### 7.1.1. Tanycytes

Tanycytes are specialized radial ependymoglial cells that line the wall of the 3^rd^ ventricle [19]. These cells have traditionally been classified into four subtypes (α_1_, α_2_, β_1_ and β_2_); however, this classification may not reflect the actual morphological and genetic heterogeneity of tanycytes, which may be influenced by numerous factors including the type of cells with which tanycytes interact [19]. 

In the ME, the cell bodies of tanycytes line the floor of the 3^rd^ ventricle, while their processes extend toward the external zone of the ME [19]. The endfeet of ME tanycytes are in contact with the parenchymal surface of the basal lamina, creating a barrier between the brain and the pericapillary space around the fenestrated capillaries of the HPS [19]. In addition, the cell bodies of adjacent ME tanycytes are interconnected with tight junctions, preventing the diffusion of circulating molecules that enter the fenestrated endothelial cells of the HPS to the CSF [354] (Figure 2, Inset 2). Dorsolateral ME tanycytes extend long processes that span through the ventromedial ARC, arching down to the pial surface, where they end at the pericapillary space or the fenestrated capillaries of the lateral external ME [19] (Figure 2). 

Ventromedial ARC tanycytes (the so-called β_1_ tanycytes) selectively express Small Proline Rich Protein 1A (SPRP1A) [19]. SPRP proteins were initially identified as keratinocyte cytoplasmic proteins that become cross-linked to membrane proteins by transglutaminase, forming the cornified cell envelope below the plasma membrane [355]. SPRPs have been used as markers of stratified squamous epithelia, where increased thickness and, at the same time, extreme flexibility are required [355]. However, SPRPs have pleiotropic functions, being involved in cell migration, EMT and prevention of DNA damage [355]. A subgroup of β1 tanycytes (which are in direct contact with fenestrated capillaries) express tight junction proteins, whereas the portion of tanycytes that skip over the capillary loops ending at pericapillary spaces lack tight junctions [354,356]. Dorsally, ARC tanycytes end at or pass by BBB vessels before entering the parenchyma of the ARC [19,354] (Figure 2, Inset 2). 

At the caudal tuberal region (the limit between the base of the hypothalamus and the hypophyseal stalk), tanycytes constitute an embryologically distinct subpopulation, expressing Shh during development [155]. These tanycytes derive from the floor of the neural plate, whereas tanycytes in the hypophyseal stalk are essentially ME tanycytes, lacking Shh expression [155]. At the dorsomedial ARC, the VMN and DMN, tanycytes also originate from Shh-expressing NPCs, and show a disorganized pattern of expression of tight junction proteins [354]. 

Dorsomedial ARC tanycytes allow the paracellular diffusion of molecules [354]. Therefore, there is no tight barrier between the dorsomedial ARC and the CSF [354] (Figure 2, Inset 2). Dorsally to the ARC and at the levels of the VMN and DMN, the cell bodies of multiciliated ependymal cells begin intermingling with tanycytes, lining the wall of the 3^rd^ ventricle and preventing the diffusion of molecules between the CSF and the hypothalamic parenchyma [354,357]. Dorsomedial ARC, VMN and DMN tanycytes are biciliated, whereas ventromedial ARC tanycytes contain only a primary cilium [357]. 

Besides the ME, tanycytes are also present in other CVOs [345]. In the CVOs, tanycytes are in contact with endothelial cells, pericytes and the ventricular wall, and express tight junction proteins, probably aiming at restricting the passive diffusion of biomolecules from the systemic circulation into the CSF, thus shifting the barrier function from the incomplete BBB (at the fenestrated endothelial cells) to the ventricular wall [335,345].

##### Tanycytes as NPCs

Tanycytes express various NPC markers, including nestin, vimentin, SOX2, GLAST, GFAP, BLBP and Musashi RNA binding protein 1 (MSI1); the expression of these markers is highly conserved across mammals [19]. In the adult hypothalamus, tanycytes retain their capacity to express genes expressed in ESCs, including Neurogenic Locus Notch Homolog Protein 1 and 2 (NOTCH1 and NOTCH2), Hes Family BHLH Transcription Factor 5 (HES5), LHX2 and RAX [19]. In addition, tanycytes express UGS148 and Serine Protease 56 (PRSS56), two proteins selectively expressed in SVZ and SGZ NPCs [19]. UGS148 is a poorly characterized protein, highly expressed in tanycytes and hypothalamic neurons [358]. UGS148 is an endoplasmic reticulum membrane protein with an intrinsically disordered domain that protrudes into the cytoplasm [358]. In addition, UGS148 associates with the mitochondrial ATPase subunit [358].

In vivo, tanycytes can proliferate, self-renew, and give rise to neurons and GFAP^+^astrocytes; however, it is unknown whether tanycytes can also generate oligodendroglial lineage cells [19]. In vitro, tanycytes are capable of generating neurospheres [19]. Tanycytes show variable NPC marker expression, proliferative properties and progeny fate [19]. During the early postnatal period, the floor of the 3^rd^ ventricle contains actively proliferating β_2_ tanycytes, which mainly generate neurons that populate the ME [19,155]. In the adult brain, tanycytes primarily generate neurons and, to a lesser extent, glial cells that populate more distant hypothalamic regions, including the ARC, VMN, DMN, posterior and lateral hypothalamus [359]. ME-ventromedial ARC FGF10^+^ β tanycytes coexpress nestin, SOX2, BLBP and MSI1 but do not express GFAP or GLAST [19]. These tanycytes generate neurons that populate the ARC and VMN but do not give rise to GFAP^+^ NPCs [347] (Figure 2, Inset 2). Dorsally, at the levels of the dmARC and VMN, a subpopulation of GLAST^+^ α tanycytes with both gliogenic and neurogenic capacity provides the ventral region within and around the VMN with astrocytes and a few neurons [348] (Figure 2, Inset 2). In vitro, α tanycytes can generate neurospheres, but β tanycytes cannot [348]. α_2_ (dorsomedial ARC) tanycytes proliferate selectively when stimulated with FGF2 (a known mitogen for SVZ NPCs), or after induced ARC neuronal death [19]. GFAP^+^ α tanycytes have the highest self-renewal capacity in vitro [348]. Since GLAST^+^ α tanycytes can generate β_1_ (ventromedial ARC) tanycytes, whereas β tanycytes are exclusively neurogenic, cannot form neurospheres, and highly express doublecortin-like (DCL), it can be deduced that GLAST^+^ α tanycytes are NPCs, whereas β tanycytes represent a more committed type of neural progenitors [19]. Of note, in the adult brain, dorso- and ventro-medial ARC tanycytes (embryologically originating from the Shh-expressing floor plate) continue to express Shh, in contrast to ME tanycytes [357,360]. Moreover, ME-ventromedial ARC tanycytes express FEZ Family Zinc Finger 2 (FEZF2), a highly conserved transcription factor involved in the patterning of the developing diencephalon [361]. FEZF2 expression may modulate the balance between quiescence and activation by guiding Notch signaling in adjacent NPCs; high FEZF2 expression reflects quiescence, whereas low FEZF2 expression is a feature of proliferation [362].

##### Τanycytes as Sensors and Integrators of Peripheral Metabolic Signals

Tanycytes sense metabolic information from the CSF and the blood, modulating neural responses accordingly [363]. Tanycytes act as glucosensors [19]. Several mechanisms through which tanycytes may sense the levels of extracellular glucose have been proposed: (i) activation of tanycytic Taste 1 Receptor Member 2 (TAS1R2) by glucose, leading to the induction of intracellular Ca^++^ currents that trigger the release of ATP from tanycytes; ATP, in turn, activates purinergic receptor P2Y1 in neighboring tanycytes, propagating Ca^++^ currents [364], (ii) activation of Monocarboxylate Transporter 2 (MCT2) in glucose-sensing ARC neurons by lactate released from tanycytes in response to changes in the levels of extracellular glucose [19,365], (iii) activation of tanycytic FGF receptors [363], and (iv) activation of Cannabinoid Receptor 1 (CB1R) in hypothalamic neurons by 2-arachidonoylglycerol released from tanycytes in response to increased levels of extracellular glucose [366]. Of note, Glucose Transporter 2 (GLUT2), glucokinase and ATP-sensitive potassium (K_ATP_) channels, which are responsible for blood glucose sensing and regulation by pancreatic cells, are also expressed in tanycytes [367]. 

During fasting-induced hypoglycemia, tanycytic VEGFA is upregulated, resulting in tanycytic-vascular remodeling in the ME-ventromedial ARC [356]. VEGFA activates VEGF receptor 2 (VEGFR2) in endothelial cells, promoting the fenestration of the endothelium of the capillary loops in the ME-ventromedial ARC, thus leading to increased vascular permeability specifically in this region [356]. This phenomenon is accompanied by the upregulation of tight junction proteins in the ME-ventromedial ARC, preventing the extravasation of biomolecules from the blood into the CSF [19]. Refeeding reverses the above effects [19]. Of note, the destruction of the wall of the 3^rd^ ventricle results in an impaired feeding response to hypoglycemia, which is reversed when tanycytes are restored [368]. 

Tanycytes serve as intermediates that regulate the transport of circulating leptin into the MBH [339]. Circulating leptin is taken up by the endfeet of ME tanycytes and is retrogradely transported to tanycytic cell bodies, being released into the CSF through transcytosis [339]. Activation of LepR and the subsequent triggering of ERK signaling are essential for the release of leptin into the CSF [339]. CSF leptin enters the ARC through paracellular diffusion at the level of ARC tanycytes, which have different polarity and barrier properties from ME tanycytes [19,339] (Figure 2, Inset 2). Through this mechanism, CSF leptin may reach other hypothalamic nuclei [19] (Figure 2, Inset 2). In addition, through the beating of the cilia of ependymal cells (a process controlled by melanin-concentrating hormone (MCH) released from the LHA), CSF leptin may be forwarded to other LepR-expressing regions in the CNS, including the hippocampus, amygdala and cerebral cortex [19,339]. The paracellular diffusion of leptin at the level of the ARC is influenced by the dynamic expression of tight junction proteins, regulated by the metabolic status of the organism [19]. 

Therefore, the metabolic status of the organism controls the permeability of the hypothalamic “neurovascular unit”—especially at the level of the ME-ventromedial ARC—to biomolecules from the systemic circulation through the fine-tuning of the fenestration of the vascular endothelium and the expression of tight junction proteins, a process that collectively preserves homeostasis and enhances organismal adaptation.

##### Tanycytes as Central Regulators of the Neuroendocrine Secretion

PVN parvocellular neurons release TRH into the fenestrated portal capillaries of the ME [155]. TRH is transported to the adenohypophysis inducing the release of TSH from the latter [155]. TSH mediates the release of T4 from the thyroid gland [155]. T4 is converted into T3 (the active form) by Iodothyronine Deiodinase 1 and 2 (DIO1 and DIO2), exerting negative feedback on TRH synthesis and release [155]. The terminals of TRH neurons end in close vicinity with the endfeet of ME tanycytes [369] (Figure 2). ME tanycytes represent the major DIO2-expressing cellular type in the CNS, regulating the conversion of T4 to T3 in the MBH [370,371,372,373]. The coordination of T4 to T3 conversion by the ME tanycytes and hypophyseal thyrotrophs is essential for normal plasma T3 levels [373]. In addition, ME tanycytes highly express Pyroglutamyl Peptidase II (PPII), an ectopeptidase that hydrolyses TRH [374], indicating that tanycytes may regulate the availability of TRH before its transport to the HPS [369]. Of note, during fasting, the levels of PPII in tanycytes transiently increase, indicating that these cells mediate the decrease of TRH levels following caloric restriction [155,375]. In addition, tanycytes express two transporters involved in the uptake of T4 and T3 (Organic Anion Transporter Family Member 1C1 (OATP1C1) and MCT8) [376]. Therefore, tanycytes may take up T4 from the blood or the CSF and convert it to T3, providing negative feedback on TRH synthesis [19,369,377]. T3 released from the endfeet of tanycytes might be taken up by adjacent TRH neuronal terminals, then retrogradely transported to the cell bodies of TRH neurons, inhibiting the transcription of TRH [369,378]. In addition, T3 released from tanycytes may directly act on the ARC, regulating energy homeostasis [369]. Food deprivation results in increased DIO2 expression in ME tanycytes and in a global increase in the levels of T3 in the hypothalamus [375,379,380,381,382]. In vivo, activation of TRH receptors type-1 (TRHR1s) results in the selective increase in the levels of intracellular Ca^++^ in ME tanycytes, enhancing the outgrowth of tanycytic processes that ensheathe TRH terminals, and increasing the activity of PPII [383]. Given the above, ME tanycyte-TRH neuron interactions may control the HPT axis [19,369].

Tanycytes, ME astrocytes and endothelial cells are involved in the tight regulation of the release of GnRH [384]. GnRH is released from hypothalamic GnRH neuron terminals in the ME and is then transported to the adenohypophysis, where it stimulates the release of luteinizing hormone (LH) and follicle-stimulating hormone (FSH) [155]. Tanycytes undergo estrous cycle-dependent morphological changes that result in the ensheathment of GnRH axon terminals by tanycytes (restricting the access of GnRH axon terminals to the perivascular space) alternating with the retraction of tanycytic processes from GnRH axon terminals (facilitating the access of GnRH terminals to the blood vessels) [385,386].

### 7.2. Humans

The existing evidence about hypothalamic neurogenesis in humans comes from a few studies based on the immunodetection of NPC markers in the postmortem brain, as lineage tracing and pulse-chase (used in animal models) are impossible in humans [16,140]. In humans, the hypothalamic neurogenic niche shows a more complex cytoarchitecture compared to that of the SVZ and SGZ, comprising at least four distinct subpopulations of NPCs: ependymal cells, ribbon cells, suprachiasmatic cells and tanycytes [16,387]. These cells coexpress nestin, SOX2, vimentin, GLAST and GFAP but differ in morphology and localization [16,387] (Figure 2). 

Ependymal cells line the wall of the 3^rd^ ventricle and express variable levels of NPC markers without any marker-specific specialized localization across the ventricular wall [387]. Most ependymal cells express high levels of SOX2 and GLAST, and low to high levels of vimentin, whereas nestin and GFAP are present only in a fraction of these cells [387]. Around 30% of ependymal cells coexpress SOX2 and nestin; ~97%, 99% and 81% of SOX2^+^/nestin^+^ ependymal cells also coexpress vimentin, GLAST or GFAP, respectively, while ~77% of SOX2/nestin^+^ cells coexpress vimentin/GLAST/GFAP [387]. Ependymal cells invaginate into the neighboring hypothalamic parenchyma forming crowns or rosettes [387] (Figure 2).

Ribbon cells constitute a population of small-sized NPCs with multiple processes, mainly expressing SOX2, vimentin, GLAST and GFAP [387]. These cells are consistently present at the border of the wall of the 3^rd^ ventricle, separated from ependymal cells by a hypocellular gap, collectively showing a gap-and-ribbon organization [387] (Figure 2). The width of the ribbon and the gap is variable [387]. The width of the ribbon depends on the number of layers of ribbon cells [387]. Around 18% of ribbon cells coexpress SOX2 and nestin, while ~84%, 88% and 81% of SOX2^+^/nestin^+^ ribbon cells coexpress vimentin, GLAST or GFAP, respectively, with ~52% of SOX2^+^/nestin^+^ ribbon cells coexpressing vimentin/GLAST/GFAP [387]. The hypocellular gap contains nestin^+^/vimentin^+^ processes, probably representing processes of ependymal or ribbon cells, and shows GLAST and GFAP immunoreactivity [387]. The gap-and-ribbon organization is interrupted at several segments of the wall of the 3^rd^ ventricle [387]. These segments have a thick border and are enriched with SOX2^+^/nestin^+^ cells that occasionally bulge into the 3^rd^ ventricle, interrupting the ependymal layer and forming highly variable (in size and shape) buds or protrusions, which often show increased expression of nestin [387] (Figure 2). Around 47% of bud cells coexpress SOX2 and nestin; ~72%, 98% and 95% of SOX2^+^/nestin^+^ bud cells coexpress vimentin, GLAST or GFAP, respectively, with ~66% of these cells coexpressing vimentin/GLAST/GFAP [387]. Buds probably represent the local proliferation of ribbon cells [387]. Of note, NeuN expression is absent in the gap-and-ribbon organization as well as in buds but can be found in distal parenchyma sites and only occasionally in the subependymal parenchyma [387]. 

Although the expression of SOX2 and nestin seems to be spatially restricted to the border of the 3^rd^ ventricle, there is a population of small-sized stellate SOX2^+^/nestin^+^ cells scattered within the SCN parenchyma, distally from the border of the 3^rd^ ventricle [387]. These cells are morphologically similar to ribbon cells but have smaller processes [387] (Figure 2). All five NPC markers (SOX2, nestin, vimentin, GLAST and GFAP) are expressed in the SCN [387]. Around 11% of suprachiasmatic cells coexpress SOX2 and nestin; ~75%, 81% and 65% of SOX2^+^/nestin^+^ cells express vimentin, GLAST or GFAP, respectively, while ~21% of these cells coexpress vimentin/GLAST/GFAP [387]. NeuN is expressed only in a low proportion of suprachiasmatic cells [387]. There is no coexpression of SOX2 in NeuN^+^ cells, in contrast to the rodent SCN, in which SOX2 is often coexpressed with neuronal markers [387].

Ki67^+^ cells are present near the border of the 3^rd^ ventricle [387]. Most of these cells coexpress the microglial marker CD68, whereas only a few of these presumably proliferating cells coexpress SOX2/nestin [387]. Among ependymal cells, ribbon cells and suprachiasmatic cells, the highest proportion of SOX2^+^/nestin^+^ cells is represented within the subpopulation of ribbon cells [387]. Specifically, ~63% of ribbon cells, ~40% of ependymal cells and ~37% of suprachiasmatic cells coexpress SOX2/nestin [387]. However, the fraction of SOX2^+^/nestin^+^ cells that coexpress GFAP or GLAST is the same among ribbon, ependymal and suprachiasmatic cells [387]. Of note, the buds are enriched with cells coexpressing SOX2/nestin, while the proportion of bud SOX2^+^/nestin^+^ cells coexpressing GLAST is higher than that of ribbon and suprachiasmatic cells [387]. 

The gap-and-ribbon organization is interrupted at the level of the tuberal region, where the wall of the 3^rd^ ventricle is lined by the cell bodies of tanycytes, which send long, radial-like processes to the ME [16,387]. Tanycytes coexpress SOX2, nestin, vimentin, GLAST and GFAP [387]. Tanycytic processes often coexpress nestin, vimentin, GLAST and GFAP; however, there may be variable proportions of tanycytic processes expressing each marker separately [387].

In human studies, the immunodetection of DCX is limited by the impact of the postmortem delay on DCX immunoreactivity [16]. However, DCX^+^ cells that resemble immature/developing neurons have been described in the human ARC, ME and VMN in congruence with animal studies [16,140]. The morphology of human hypothalamic DCX^+^ cells is variable and location-dependent, including round-shaped cells in the subependymal zone, fusiform cells in the ventral ARC, and unipolar or bipolar cells in the VMN [140] (Figure 2). However, it is unknown whether this morphological variability represents different maturational stages and whether it is functionally meaningful [16]. If a correlation between cellular morphology and maturational stage can be extrapolated from the SVZ and SGZ (where immature neuroblasts have no or short processes, migrating neuroblasts are elongated and have a process that leads their migrational pathway, and integrating neurons have a more complex morphology), then round-shaped, fusiform, and unipolar/bipolar DCX^+^ cells (in the hypothalamic niche) might represent immature neuroblasts, migrating neuroblasts, and integrating neurons, respectively [387]. Hence, DCX^+^ cells that are generated close to the wall of the 3^rd^ ventricle may progressively migrate into the neighboring parenchyma [16]. This scenario would be in congruence with evidence from animal studies showing that tanycytes give rise to neurons that migrate into the neighboring hypothalamic parenchyma [16]. Collectively, the simultaneous detection of proliferating NPCs and DCX^+^ neurons in the adult human hypothalamus is indicative of the existence of a hypothalamic neurogenic niche [16].

In humans, the organization and marker expression at the wall of the 3^rd^ ventricle is similar to those in the SVZ, in which there is a ribbon of small process-bearing cells coexpressing nestin, SOX2, vimentin, GLAST and GFAP, separated from the ependymal layer by a hypocellular gap, which is enriched with GLAST and GFAP [387]. In the SVZ, ependymal cells coexpress SOX2 and GLAST as well as variable levels of vimentin, nestin and GFAP; this expression profile is consistent at different rostrocaudal and dorsoventral levels of the LVs [387]. Yet, the SVZ shows a more uniform organization, a more regular ribbon layer and a thinner gap compared to the wall of the 3^rd^ ventricle [387]. In addition, crowns and rosettes of ependymal cells are not present in the SVZ; however, buds similar to those found in the wall of the 3^rd^ ventricle are present in the SVZ [387]. Moreover, nestin immunoreactivity is much rarer in ependymal cells in the wall of the LVs than in the ependymal cells lining the wall of the 3^rd^ ventricle, while strong vimentin immunoreactivity is consistently present in the former [387].

### 7.3. Comparison of the Adult Human and Animal Hypothalamic Neurogenic Niches

The organization of the human hypothalamic neurogenic niche is significantly different from its rodent counterpart [16,387]. In rodents, there are no ribbon cells and no gap-and-ribbon organization at the border of the wall of the 3^rd^ ventricle [16,387]. Of note, there are differences even between rodent species; in mice, there is a regular layer of ependymal cells, whereas, in rats, the border of the 3^rd^ ventricle is irregular with thickenings and evaginations of the ependymal layer that frequently protrude into the dorsal part of the 3^rd^ ventricle [387]. Importantly, in rodents, ependymal cells do not express NPC markers [16]. By contrast, in humans, ependymal cells express all five NPC markers, which are also expressed in ribbon cells [387]. In humans, ependymal and ribbon cells are organized in a gap-and-ribbon conformation similar to that described in the SVZ [387]. Of note, ependymal cells in the human hypothalamic niche and the SVZ show different marker expression patterns; the former highly express nestin with the majority of them coexpressing GFAP, whereas the latter express low levels of nestin and rarely GFAP [387]. In addition, in the human SVZ, the ribbon contains GFAP^+^ astrocytes, capable of proliferating in vivo and exhibiting NPC properties in vitro [388].

In rodents, in the hypothalamic parenchyma around the 3^rd^ ventricle, there are scattered cells that express various combinations of NPC markers: SOX2^+^/NG2^+^, SOX2^+^/NG^2−^ and SOX2^−^/NG2^+^ cells [16]. SOX2^+^ cells can generate neurons, astrocytes and oligodendrocytes, whereas NG2^+^ cells can give rise primarily to oligodendrocytes and a few neurons [16,351]. SOX2, nestin and vimentin are coexpressed only in tanycytes [387]. In humans, SOX2 is mainly expressed near the wall of the 3^rd^ ventricle [16]. Tanycytes are the only NPC population common to the human and rodent hypothalamic niche and are conserved across numerous species [16]. However, there is limited evidence about the properties and functions of tanycytes in the human hypothalamus; as tanycytic processes can express different combinations of NPC markers, such as nestin/vimentin, nestin/GLAST or nestin/GFAP, or even each marker separately, tanycytes might represent molecularly and functionally heterogeneous subpopulations of NPC-like cells [16,387]. 

In the adult rodent SCN, several NPC and neuroblast markers, such as SOX2, DCX, doublecortin-like (DCL), NeuN and PSA-NCAM, are expressed; however, NPC-like cells or neurogenesis have not been reported [16,387]. In the adult rodent SCN, SOX2^+^ cells do not coexpress nestin or vimentin [16,387]. By contrast, in the adult human SCN, suprachiasmatic cells express NPC markers but do not express NeuN [387]. In the rodent hypothalamus, SOX2 is thought to be involved in the regulation of the expression of CLOCK genes, neuropeptides and neuropeptide receptors [389]. However, recent evidence showed that, in the embryonic rodent SCN, SOX2 acts as a differentiation factor [390]. Ablation of SOX2 during early SCN development disrupts AVP and VIP expression before the downregulation of LHX1 and SIX Homeobox 6 (SIX6), compromising the ability of neuropeptidergic neurons to survive during the postnatal cell clearance window [390]. 

Interestingly, the adult human and sheep hypothalamus show similar DCX expression patterns with similar morphology and distribution of DCX^+^ neuroblasts, whereas there are only sparse DCX^+^ cells in the adult rodent hypothalamus [140]. The functional significance of this discrepancy in the distribution of DCX^+^ cells in different species remains unknown [140]. It has been proposed that the origin of DCX^+^ cells in rodents might be ME tanycytes or another not yet specified NPC subpopulation [140].

### 7.4. Regulation and Functional Implications of Adult Hypothalamic Neurogenesis

#### 7.4.1. Energy Balance and Metabolism

##### HFD impairs Neurogenesis in the ARC, similarly to Leptin Deficiency

In adult mice, considerable cellular turnover takes place in the ARC, resulting in continuous neuronal remodeling [391]. Long-term HFD results in fewer newly generated neurons, maintenance of old neurons, and increased apoptosis of newborn neurons in the ARC [391]. Similarly, leptin deficiency leads to fewer newborn neurons, partially attributed to the loss of hypothalamic NPCs.

##### HFD induces ARC-ME Inflammation, Neuronal Injury and Gliosis

In the ARC-ME complex of rats and mice, HFD induces the upregulation of proinflammatory genes, including IL6, TNFα, Suppressor of Cytokine Signaling 3 (SOCS3), Inhibitor of Nuclear Factor Kappa B Kinase Subunit Beta and Epsilon (IKBKB and IKBKE) [392]. This effect occurs within the first seven days of HFD initiation, before a significant weight gain, and is initially transient [392]. The expression levels return to baseline after 7–14 days but increase permanently by day 28 [392]. Neuronal injury and reactive gliosis are observed in the ARC-ME within the first week, followed by a window of temporary remission; however, upon HFD continuation, MBH gliosis is permanently reinstalled [392]. The reactive gliosis induced by HFD is characterized by the recruitment of astrocytes, formation of astrocytic syncytia, and microglial proliferation and activation, similar to the effects of ischemia or excitotoxicity [392]. After eight months of exposure to HFD, the number of ARC POMC neurons decreased by 25% compared to controls [392]. Congruently, there is evidence supporting the presence of MBH gliosis in obese human subjects, indicating that obesity is essentially associated with MBH injury [392].

##### HFD → ↑ NFκB Pathway → MBH Inflammation → Impaired MBH Neurogenesis and NPC Survival → Overeating, Weight Gain, Glucose Intolerance and Hyperinsulinemia

Long-term HFD induces the activation of the IκB kinase (IKKβ)/nuclear factor-κB (NFκB) pathway in the MBH, impairing the proliferation and survival of NPCs [349]. Activation of the NFκB pathway increases the apoptosis of NPCs through the upregulation of apoptotic genes, impairs the differentiation of NPCs into neurons (including POMC and NPY neurons), astrocytes and oligodendrocytes, and increases the number of microglial cells, increases the glia/neuron ratio and upregulates Tumor Necrosis Factor alpha (TNFα) [349]. Inhibition of the NFκΒ pathway rescues the apoptosis of NPCs [349]. Thus, (a) HFD induces MBH inflammation, at least partially mediated by microglia, (b) MBH NPCs are vulnerable to local inflammation, and (c) activation of the NFκB pathway disrupts the differentiation of MBH NPCs [349]. In an adult mouse model with IKKβ/NFκΒ gain-of-function NPCs in the MBH, there was an overall 60% reduction in the number of SOX2^+^ NPCs and a 10% decrease in the number of POMC^ARC^ neurons compared to controls [349]. At three months, these mice developed glucose intolerance and hyperinsulinemia accompanied by overeating and progressive weight gain, resulting in severe obesity at ten months [349]. The activation of the NFκB pathway was transferrable to the progeny of these mice, presumably through the paracrine activation of the NFκΒ pathway by TNFα and IL-1β [349].

##### MBH NPC Ablation → Overeating, Weight Gain and Glucose Intolerance

Similarly to chronic HFD, ablation of NPCs in the adult mouse MBH results in overeating, weight gain and glucose intolerance [393]. As already mentioned, HFD induces MBH hypothalamic inflammation due to the activation of the NFκB pathway [349]. When NPCs are implanted in mice with HFD-induced obesity, they are not able to survive within the HFD-altered hypothalamic microenvironment, failing to reverse the phenotypic effects of HFD [349,393]. By contrast, when engineered NPCs, in which the NFκB pathway has been inhibited, are implanted in HFD-fed obese mice, the survival rates of these NPCs dramatically increase, reducing the phenotypic effects of HFD, including increased food intake, weight gain, glucose intolerance and hyperinsulinemia [393]. These NPCs can undergo neurogenesis, induce hypothalamic neuropeptides such as POMC, CART, AgRP and NPY, and interact with the host hypothalamic neurons [393]. In addition, the control of leptin signaling on feeding is restored [393]. Engineered NPCs influence GABAergic and glutamatergic signaling in the host hypothalamus and can give rise to at least two types of neurons: POMC^+^ and glutamic acid decarboxylase 67 (GAD67)^+^ neurons; GAD67 is an enzyme that metabolizes glutamate into GABA and is the hallmark of GABAergic neurons [393,394]. POMC^+^ and GABAergic neurons may have independent and combined effects on body weight regulation [393,395]. However, it is unknown whether the effects exerted by implanted, engineered hypothalamic NPCs are the result of the neurotransmitter/neuropeptide signaling mediated by the neurons generated by these NPCs, or by the secretome of the NPCs themselves. Interestingly, activation of the NFκB pathway leads to a programmatic switch from neurogenesis to gliogenesis, a known effect of the Notch signaling pathway [393,396]. Indeed, the effect of NFκΒ activation on neurogenesis is mediated by Notch activation [349]. 

#### 7.4.2. Sleep

##### ↓ Hypothalamic Neurogenesis → Disrupted Sleep Architecture (Aging-reminiscent)

Ref. [397] showed that intraventricular infusion of AraC (an antimitotic agent known to suppress hypothalamic neurogenesis) for four weeks dramatically decreased the number of BrdU^+^ cells around the wall of the 3^rd^ ventricle and the neighboring anterior hypothalamic area (including the MPO and SCN). This effect was phenotypically associated with sleep architecture reminiscent of that in aged mice [397]. Hence, hypothalamic neurogenesis may have a role in sleep-wake organization and circadian rhythms, with impaired hypothalamic neurogenesis potentially contributing to sleep-wake cycle dysregulation during aging [397].

#### 7.4.3. Aging

##### Aging → MBH Microglial Activation → ↑ NFκB Pathway in Neighboring Cells

In the hypothalamus of young mice, the NFκB pathway is inactive but is progressively activated from middle age onwards [398]. In the MBH of aging mice, there is a significant age-dependent increase in the number of microglial cells, accompanied by increased expression of TNFα and activation of the NFκΒ pathway [398]. During the early stages of aging, NFκΒ activation is limited to hypothalamic microglia, whereas as aging progresses, the NFκB pathway also becomes activated in neighboring cells [398]. It has been proposed that, during early aging, hypothalamic microglial soluble TNFα acts in a paracrine fashion, activating the IKKβ/NFkΒ pathway in neighboring cells, including neurons [398]. In middle-aged mice, inhibition of the NFκB pathway in the MBH results in the retardation of aging and increases their lifespan, whereas activation of the NFκB pathway results in the deterioration of systemic aging markers, including cognition, bone mass, muscle size and skin thickness [398]. IKKβ knockout in mouse MBH microglial cells prevents the age-related increase in the number of hypothalamic microglial cells and the activation of the NFκΒ pathway in neighboring cells, slowing down systemic aging [398]. Activation of the IKKβ/NFκB pathway inhibits the expression of hypothalamic GnRH by decreasing GnRH promoter activity; this effect is reversed by NFκΒ inhibition [398]. Intraventricular delivery of GnRH promotes adult neurogenesis throughout the brain despite aging [398]. Similarly, peripheral administration of GnRH reduces the histological effects of systemic aging and reverses age-associated cognitive decline [398]. 

##### Loss of SOX2/BMI1^+^ Cells in the Wall of the 3^rd^ Ventricle at the Level of MBH drives Aging

In adult mice, the maintenance of MBH SOX2^+^/Polycomb complex protein BMI-1 (BMI1)^+^ NPCs reduces hypothalamic inflammation and systemic aging, whereas their loss is a significant cause of systemic aging [399]. In numerous physiological systems, including the CNS and PNS, BMI-1 is essential for the postnatal self-renewal and maintenance of stem cells [400]. BMI-1 represses the transcription at the INK4a-ARF locus [400]. INK4a encodes p16INK4a, a cyclin-dependent kinase inhibitor that activates Rb [401]. ARF encodes p19ARF, which activates p53 [400]. Both Arb and p53 are inhibitors of cell proliferation [401]. p16INK4a and p19ARF expression can cause cellular senescence in vitro [402]. In young mice, SOX2^+^/BMI1^+^ cells are densely distributed in the wall of the MBH portion of the 3^rd^ ventricle and are sporadically present in the parenchyma of the MBH [399]. The number of SOX2^+^/BMI1^+^ cells gradually decreases as age progresses from middle age onwards [399]. Selective ablation of SOX2^+^/BMI1^+^ cells results in a significant decrease in the lifespan compared to control animals [399]. Hence, the wall of the 3^rd^ ventricle at the level of the MBH is crucial for the regulation of the speed of aging [399]. Transplantation of neonate mice-derived hypothalamic NPCs into the MBH of middle-aged mice results in the destruction of these NPCs because of the already established MBH inflammation in middle-aged mice; however, blockade of the NFκB pathway results in a dramatic increase in the survival of these NPCs [399]. Implantation of neonate mice-derived hypothalamic NPCs with blocked NFκΒ in the MBH of aged mice results in significant beneficial systemic effects independently of the pattern of food intake [399]. These effects are superior to those of the implantation of astrocytes or mesenchymal stem cells (MSCs) and are initially observed six weeks after implantation [399]. Hypothalamic NPCs are markedly enriched with exosomes (compared to hypothalamic astrocytes, GT1-7 cells and MSCs) [399]. Hypothalamic NPC-derived exosomes contain ~100-fold more miRNAs than hypothalamic astrocyte-derived exosomes; hypothalamic NPCs exchange miRNAs through exosomal delivery [399]. In addition, miRNAs originating from hypothalamic NPCs are present in the CSF [399]. Inhibition of exosomal biogenesis/release decreases CSF miRNA levels [399]. Moreover, CSF miRNA levels decline with aging [399]. It has been proposed that hypothalamic NPC-derived exosomal miRNAs and neuropeptides released from these cells induce anti-inflammatory and anti-aging effects in a paracrine/endocrine fashion [399].

##### Hypothalamic NPCs can form Spheres with Combined Prosurvival and Antidiabetic Effects

In mice, subpopulations of hypothalamic NPCs can form 3D spherical structures with combined features of neurospheres and pancreatic islets, synthesizing various hypothalamic, pancreatic and gastrointestinal peptides as well as exosomes [402]. In mice, peripheral implantation of these spheres results in prosurvival and antidiabetic effects [402]. Interestingly, exosome release from these spheres is essential for the prosurvival effect, whereas insulin production is necessary for the antidiabetic effect, with prosurvival and metabolic effects being significantly separable [402].

#### 7.4.4. Temperature/Heat Acclimation

Exposure of rodent hypothalami to moderate heat for six days resulted in increased numbers of BrdU^+^ cells [403]. Most of these cells initially appeared in the ventricular zone of the 3^rd^ ventricle, progressively becoming present in the parenchyma [403]. Following 33 days of heat exposure, the number of NeuN^+^/BrdU^+^ cells was 7-fold higher in the hypothalamus of heat-exposed rodents than in controls [403]. Such cells were present in the VMN, DMN, anterior hypothalamus and preoptic area (POA) [403]. In the POA, heat stress-induced BrdU^+^ cells expressed markers of GABAergic and glutamatergic neurons and showed increased c-fos expression [404,405]. Inhibition of cell proliferation disrupted heat acclimation [403,404]. Thus, moderate heat stress may increase neurogenesis, which, in turn, may result in the modification of the hypothalamic neurocircuitry leading to long-term changes in thermoregulation [8]. The central node of the neurocircuitry that controls body temperature is the POA, although other hypothalamic nuclei, such as the PVN and DMN, are involved [8]. POA neurons control body temperature through tonic GABAergic signaling [405]. Heat acclimation lowers basal body temperatures and results in a slower increase in body temperature following heat exposure than in naïve individuals [406,407]. In the DG, short-term heat exposure promotes adult neurogenesis through AT1Rs activation [408]. However, there is also evidence that, in the hippocampus of adult mice, heat stress may activate glial cells and proinflammatory mediators, inducing loss of neurons and synapses, resulting in disrupted neurogenesis, which is associated with cognitive deficits [409]. Hence, it is plausible that the effects of heat exposure on adult hypothalamic neurogenesis follow an upside-down U-shaped curve, resembling the Yerkes–Dodson curve of pressure and performance [410], a general principle that delineates the effects of stressors on organismal homeostasis.

#### 7.4.5. NE

NE is a negative regulator of neurogenesis in the adult caudal periventricular neurogenic niches (i.e., the hypothalamic and midbrain aqueduct neurogenic niches) but a positive regulator of neurogenesis in the SGZ [411]. In the CNS, most NE originates from the LC, which projects virtually to all CNS regions [236]. In the SVZ, low NE levels (presumably due to sparse afferent projections from the LC) correlate with high NPC proliferation capacity [411]. By contrast, in the caudal periventricular niches, high NE levels (due to dense afferent projections from the LC [236]) are associated with low NPC proliferation capacity [411]. NE suppresses the proliferation of NPCs by promoting their exit from the cell cycle and their differentiation into neurons, probably through the activation of β-adrenergic receptors in NPCs [411]. The pharmacological blockade of NE promotes the proliferation of adult NPCs and early neurogenesis in the hypothalamic and midbrain periventricular neurogenic niches [411]. Of note, NE antagonism does not alter the proportion of vimentin^+^ tanycytes but increases other hypothalamic NPC populations, including NG2^+^ glial cells, SOX2^+^/GFAP^+^ parenchymal astrocytes and DCX^+^ neuroblasts [411]. In congruence with this finding, NE antagonism mediates the proliferation of NPCs mainly in the subventricular/subependymal parenchyma directly adjacent to the tanycytic layer in the lateral wall of the 3^rd^ ventricle close to the DMN, VMN and ARC, but not in the ME [411]. Similarly, the SGZ receives dense innervation from the LC [236,412,413]. In the SGZ, NE positively regulates neurogenesis through β2 or β3 adrenergic receptors expressed in NPCs [414,415]. Remarkably, in the SGZ, NE depletion results in a swift (after a few days) decrease in the rate of NPC proliferation but does not affect the long-term survival and differentiation of NPCs after 4–9 weeks [411]. Therefore, in the SGZ, NE initially stimulates NPC proliferation but does not affect the net NPC survival or neuronal differentiation [411]. 

#### 7.4.6. Transcription Factors of the NFI Family

In the postnatal mouse hypothalamus, tanycytes retain a latent capacity to generate a wide range of different subtypes of hypothalamic neurons; however, transcription factors of the Nuclear factor I (NFI) family—highly expressed in hypothalamic tanycytes and glial cells—repress this capacity [416]. Of note, α_2_ tanycytes generate proliferating tanycytes, which give rise to neural precursors after they exit the cell cycle, whereas astrocytes originate directly from α_1_ and α_2_ tanycytes without an intermediate proliferating stage [416]. Selective loss of function of NFIα/b/x in tanycytes induces the regression of these cells to a progenitor-like state, increasing their proliferative and neurogenic competence and leading to the abundant generation of hypothalamic neuronal precursors that undergo outward radial migration and maturation and are capable of receiving synaptic inputs and generating action potentials. These effects are associated with the downregulation of components of the Notch pathway and TGFβ2 and upregulation of Delta-like non-canonical Notch ligand 1 (DLK1) (a Notch inhibitor). In addition, SOX8 and SOX9 (required for the specification of astrocytes) are downregulated, whereas SOX4 and ASCL1 (required for neurogenesis) are upregulated [416].

Tanycyte-derived neurons arise from ASCL1^+^ precursors and are heterogeneous, constituting distinct neuronal clusters [416]. These neurons are mostly GABAergic and express molecular markers of ARC, DMN and VMN neurons [416]. Tanycyte-derived neurons progressively mature and survive for months [416]. However, these neurons generate fewer action potentials than pre-existing neighboring neurons, indicating that the former may be inherently less efficient in forming synaptic connections than the latter [416]. Tanycyte-derived neurons include neuronal subtypes that regulate feeding, sleep and pituitary function; however, some remain poorly characterized [416]. Tanycyte-derived neurons respond to dietary and environmental signals, such as leptin and heat stress [416]. Hence, the physiological milieu may influence the generation and survival of distinct subsets of tanycyte-derived neurons, modifying hypothalamic circuits [416].

## 8. Discussion

The structural and functional organization of the hypothalamus is remarkably conserved in vertebrates, indicating that hypothalamic induction, patterning and neurogenesis may be underpinned by conserved molecular mechanisms [173]. Yet, species-specific developmental programs may result in evolutionarily preserved or extinct modules that comprise essential (thus, conserved) or dispensable (thus, lost due to the lack of selective pressure) neural networks [173]. 

Tanycytes are the only type of NPC-like cells common to humans and rodents [16]. In rodents, tanycytes constitute four distinct subpopulations with variable neurogenic potential, widely distributed at different levels of the wall of the 3^rd^ ventricle [16,19]. By contrast, in humans, tanycytes are restricted to the wall of the floor of the 3^rd^ ventricle, a distribution corresponding to that of β_1_ and β_2_ tanycytes in rodents [16,19]. The morphological, molecular and functional characterization of tanycytes remains elusive [16,19]. In humans, besides tanycytes, there are three additional distinct subpopulations of NPC-like cells: (i) ependymal cells, (ii) ribbon cells and (iii) suprachiasmatic cells [19,387]. In humans, ependymal cells line the wall of the 3^rd^ ventricle at various levels but the floor, and express stemness markers, whereas in rodents ependymal cells have no neurogenic potential [16,19,387]. In humans, at the border of the wall of the 3^rd^ ventricle, there is a ribbon of small process-bearing NPC-like cells, separated from ependymal cells by a hypocellular gap containing ependymal or ribbon cell processes; this gap-and-ribbon organization is similar to that present in the SVZ [16,387]. In humans, the ependymal layer is sporadically interrupted by buds that protrude into the 3^rd^ ventricle and are highly enriched with SOX2^+^/nestin^+^ cells [387]. Buds presumably represent the local proliferation of ribbon cells [387]. In humans, NPC-like cells that resemble ribbon cells are present in the parenchyma of the SCN [387]. Neuroblasts have been identified in the postmortem adult human hypothalamus, primarily at distant parenchymal sites (especially the ARC and VMN), and only occasionally in the subependymal parenchyma mainly at the floor of the 3^rd^ ventricle. However, DCX staining is limited by postmortem delay [16,387].

The above findings have generated the following questions: (a) Can adult hypothalamic ependymal cells indeed proliferate and differentiate? (b) What is the trajectory, progeny and functional role of these cells? (c) Why do hypothalamic ependymal cells express NPC markers in humans but not in rodents? Hypothalamic ependymal cells are situated in a key position, with one surface facing the hypothalamic parenchyma and another surface facing the CSF. One wonders whether adult hypothalamic ependymal cells contribute to the regulation of the hypothalamic and extrahypothalamic neurogenic niches and neural circuits in a paracrine and endocrine fashion, respectively, through the release of signaling molecules such as neuropeptides, neurotransmitters, growth factors, ATP and non-coding RNAs (ncRNAs), and EVs. Adult hypothalamic ependymal cells may convey signals from the hypothalamic parenchyma, through the CSF, to extrahypothalamic sites. In the opposite direction, hypothalamic ependymal cells may transfer cues from extrahypothalamic sites to hypothalamic nuclei. Therefore, hypothalamic ependymal cells may contribute to the complex interactions between hypothalamic and extrahypothalamic neurogenic niches and neural circuits. Since ependymal cells have neurogenic potential in the adult human hypothalamus but not in the rodent one, one wonders whether these cells are involved in intercellular and internetwork communication associated with high-order functions and behaviors specific to humans. Interestingly, in the adult human hypothalamus (but not in the SVZ), ependymal cells invaginate into the neighboring hypothalamic parenchyma forming crowns and rosettes [387]. In addition, buds of ribbon cells interrupt the layer of ependymal cells, protruding into the 3^rd^ ventricle [387]. One wonders whether the existence of these conformations is stochastic or whether they serve a distinct functional role, e.g., they might represent sites of intense exchange of stemness-related or other signals between the hypothalamus and extrahypothalamic circuits through the CSF.

Ref. [388] described the presence of a ribbon of small-sized stellate astrocyte-like NPCs (termed SVZ astrocytes) in the human SVZ [388]. These cells are separated from the ependymal layer by a hypocellular gap consisting of GFAP^+^ processes, and coexpress GFAP and vimentin, while some of these cells also express Ki67 [388]. Some of these astrocyte-like NPCs have a process extending into the ependyma towards the lumen of the LVs [388], a similar conformation to that of hypothalamic ribbon cells described in Ref. [387]. SVZ astrocyte-like NPCs can divide in vivo (in the absence of external growth factors), generate multipotent self-renewing neurospheres in vitro, and differentiate into astrocytes, neurons and oligodendroglia [388]. In vitro, SVZ astrocyte-like NPCs can give rise to colonies of bipolar or multipolar cells that express neuronal markers [388], similar to the neuroblasts described in the adult human hypothalamus [140]. Given that the ribbon cells described in the adult human hypothalamus [387] show a similar morphology and marker profile to the SVZ astrocytes described in Ref. [388], it could be deduced that ribbon cells may represent astrocyte-like NPCs and that the bipolar or multipolar DCX^+^ cells described in Ref. [140] originate from ribbon cells. Hence, hypothalamic ribbon cells may represent pivotal NPC marker^+^ cells with pleiotropic functions, including the capacity to generate hypothalamic neurons and to regulate neurogenesis and neural circuits in extrahypothalamic sites through the CSF.

In the adult rodent hypothalamus, neurogenesis takes place mainly in the MBH [16], a region primarily involved in the regulation of primitive functions and behaviors essential for survival, such as feeding, body weight and aggression. Different subpopulations of tanycytes with variable neurogenic potential behave as NPCs [19]. In the adult human hypothalamus, tanycytes constitute only a subportion of the hypothalamic niche [387]. We propose that the tanycytic subniche represents a conserved subportion of the hypothalamic neurogenic niche that contributes to the renewal of neurons involved in low-order functions and behaviors essential for organismal survival. Nonetheless, human beings are characterized by complex behaviors effectuated by the interactions of sophisticated neural networks. We propose that, in the adult human hypothalamus, ependymal, ribbon, and suprachiasmatic cells are responsible for the renewal and plasticity of these networks.

Although the structure and function of the hypothalamus are highly conserved in vertebrates [173], the organization of the PVN differs considerably even across closely related species [296,297]. The PVN is involved pleiotropically in physiological processes, including the neuroendocrine stress response and autonomic regulation [66,157]. The PVN is reciprocally connected with the LC, constituting the two basic nodes of the stress system [66,157,236]. One wonders whether neurogenesis occurs in the adult human and animal PVN and LC (and other sites of the stress system), as well as what is the impact of the neuroendocrine stress on hypothalamic neurogenesis. In addition, it would also be worth exploring whether neurogenesis occurs in the adult human SCN and the role of such neurogenesis. Due to the limitations of the study of hypothalamic neurogenesis in the postmortem human brain [16], we propose the experimental utilization of in vitro 3D models (i.e., brain and/or hypothalamic organoids/assembloids) [157,417]. Of note, such models have been used mainly in studies on embryonic neurogenesis and neurodevelopmental disorders [157].

PACAP released from SVZ and SGZ NPCs acts as a potent NPC proliferator through the autocrine and paracrine activation of PAC1 [97,418]. The PACAP/PAC1 system is widely and highly expressed in the hypothalamus, the brain region with the highest expression of PACAP [418,419,420]. VPAC1 is not expressed in the hypothalamus, while VPAC2 shows medium-low expression [421]. PACAP and PAC1 are highly expressed in PVN neurons [421]. One wonders whether PACAP and/or its receptors are expressed in adult human hypothalamic NPC subpopulations. We propose that the PACAP/PAC1 system plays a vital role in the hypothalamic neurogenic niche, as well as in the regulation of extrahypothalamic neurogenic niches and neural circuits by the hypothalamus. As the PVN shows high expression of PACAP/PAC1 [418,419,420], neurogenesis might indeed occur in the PVN.

Within the PVN, circuits of GABAergic and glutamatergic interneurons integrate the neuronal input with the neuroendocrine/autonomic output [314]. In addition, a distinct subpopulation of CRHR1^+^ neurons (activated by CRH released from CRH neurons) sends GABAergic projections back to CRH neurons, forming a regulatory feedback loop [315,316]. These intraPVN interneuronal networks are reminiscent of the local circuits present in the SGZ, which couple neuronal activity with the NPC quiescence/activation balance [79]. We propose that the PVN subniche may be the structural analog of the SGZ. In the adult DG, VIP coreleased with GABA from GABAergic interneurons activates VPAC2 in NPCs, promoting their proliferation [93]. VIP and GABA are coexpressed in SCN neurons that project to CRH^PVN^ and descending^PVN^ neurons, as well as to the SPZ [273,281]. One wonders whether VIP/GABA^SCN^ neurons also project to hypothalamic NPCs and whether there is any specialized localization of these NPCs. On the other hand, the PVN CRH/CRHR system is reminiscent of the local CRH/CRHR system expressed in mouse and human hippocampal NPCs; this system is essential for hippocampal neurogenesis [327]. One wonders whether there is a PVN subniche, in which NPCs express CRHRs activated by the local release of CRH. Overall, the structural, cellular and molecular profile of the PVN is indicative of the existence of a neurogenic niche within the PVN, resembling the SGZ.

We propose that the human hypothalamic niche is multifaceted and highly sophisticated, showing structural and functional compartmentalization. (A) The “low-order” highly conserved tanycytic subniche is responsible for the maintenance of neurogenesis in primitive hypothalamic centers involved in feeding and metabolism; through this subniche, peripheral signals reach tanycytes through the ME. (B) The gap-and-ribbon subniche (formed by ribbon and ependymal cells) resembles and may actually constitute the hypothalamic structural analog of the SVZ. As this subniche is widely distributed across the wall of the 3^rd^ ventricle, it may be responsible for the overall regulation of the hypothalamic niche, as well as the control of extrahypothalamic neurogenic niches and neural circuits by the hypothalamus through the CSF. Ribbon cells may play a pivotal role in this subniche, representing the cellular analogs of the SVZ astrocyte-like NPCs described by Ref. [388]. (C) The “high-order” PVN subniche is characterized by the presence of interneuronal glutamatergic/GABAergic circuits, resembling those in the SGZ; thus, this subniche may constitute the hypothalamic structural analog of the SGZ. The PVN subniche may be responsible for the regulation of neurogenesis in response to neuroendocrine and autonomic signals. (D) The SCN subniche consists of ribbon cell-like cells, which may represent modified astrocyte-like NPCs. This neurogenic subniche might be responsible for the regulation of neurogenesis by biological rhythms. One wonders whether and how the hypothalamic subniches (i) interact with each other and (ii) are regulated by hypothalamic and extrahypothalamic circuits and their effectors.

The hypothalamus is a brain region where neurotransmitter and neuropeptide signaling are remarkably intertwined; neurotransmitters mediate primarily the control of extrahypothalamic networks over hypothalamic nuclei, whereas neuropeptides effectuate intrahypothalamic communication and the neuroendocrine output, while they modulate extrahypothalamic networks. Remarkably, neurotransmitter signaling within and across various hypothalamic nuclei is involved in intrahypothalamic communication, as well as in the interactions of hypothalamic nuclei with extrahypothalamic sites.

Through glutamatergic signaling, the DMN stimulates LHA orexinergic and non-orexinergic neurons [273]. Glutamatergic signaling is prominent in the VMN, being part of a neurocircuitry that regulates glucose metabolism and the CRR [238,246]. In addition, VMN glutamatergic projections to the PAG and brainstem effectuate aggression associated with survival [238]. RHT glutamatergic projections provide photic input to the SCN [281]. In the PVN, glutamatergic projections from extrahypothalamic neuronal networks stimulate CRH neurons mediating neuroendocrine stress [66]. In addition, glutamatergic intraPVN interneurons mediate the stimulatory effect of various hypothalamic and extrahypothalamic circuits on the PVN [314].

GABAergic^DMN^ neurons project to the VLPO and LC, modulating the balance between sleep and wakefulness/arousal, and to NPY/AgRP^ARC^ and POMC^ARC^ neurons [273]. Food presentation induces the activation of LepR/GABA^DMN^ neurons that inhibit NPY/AgRP^ARC^ neurons [422]. GABA is coreleased from NPY/AgRP^ARC^ neurons, which inhibit POMC/CART^ARC^ neurons, regulating food intake and glucose metabolism [195,422]. Of note, GABA is also coreleased from POMC/CART^ARC^ neurons; however, the role of this release remains unclear [422]. GABA is released from first-order leptin-sensing NPY^−^/AgRP^−^ and POMC^−^ GABAergic^ARC^ neurons [422]. Disrupted GABA release from hypothalamic RIP^+^ neurons results in obesity characterized by dysregulated energy expenditure but normal food intake [422]. GABA is coreleased from MCH^LHA^ neurons [422]. Single-cell RNA-seq showed that the hypothalamus contains fifteen clusters of GABAergic neurons, including neurons that express AgRP/NPY, POMC, somatostatin and CRH, and that these clusters represent sites of GABA/dopamine coexpression [422,423]. According to another single-cell RNA-seq study, GABAergic^ARC/ME^ neurons can be classified into eighteen clusters, including somatostatin^+^ and somatostatin^−^ AgRP neurons [198]. Interestingly, a subpopulation of somatostatin^+^/GABA^+^/AgRP^−^ neurons are transcriptionally similar to AgRP neurons; activation of the former induces food intake, similarly to the activation of the latter [198]. Of note, no GABAergic neuronal subtype that suppresses feeding has been reported so far [422]. In the SCN, GABA is a ubiquitous neurotransmitter coexpressed with neuropeptides in all subpopulations of SCN neurons [281]. The VIP/GABA balance modulates the intraSCN synchrony, while VIP^SCN^ neurons may inhibit various groups of target neurons (including CRH^PVN^, descending preautonomic^PVN^ neurons, SPZ and VMN neurons) via synchronized waves of GABAergic rather than VIPergic activity; the relative contributions of the GABAergic versus VIPergic SCN output seems to be variable across species [281]. GABAergic interneurons are located in a halo around the PVN, regulating the excitability of PVN neurons [314]. A specific subpopulation of GABAergic interneurons express CRHR1s stimulated by CRH released from CRH^PVN^ neurons, creating an autoinhibitory feedback loop [315,316]. 

Hypothalamic neurotransmitter/neuropeptide co-signaling may provide an ideal physiological milieu for a highly complex and flexible neurogenic niche, regulated by the balance between classical neurotransmission (through GABA/glutamate interneuronal networks) and the actions of a remarkable variety of modulatory neuropeptides. The latter may regulate local or/and distal, mature or/and immature neural networks, as well as local or/and distant NPC populations, through autocrine and paracrine signaling (local networks), volume transmission (distal networks), as well as through the CSF (extrahypothalamic networks). Hence, the hypothalamus may be the master regulator of hypothalamic and extrahypothalamic neurogenesis and neural circuits. 

The hypothalamus liaises between the periphery of the organism and the CNS through the CVOs, which provide controlled access to selected metabolic, inflammatory and autonomic signals; these signals might modulate hypothalamic and extrahypothalamic neurogenesis (through the hypothalamus). Therefore, modulation of peripheral signals might be a means of influencing hypothalamic and extrahypothalamic neurogenesis. In the opposite direction, hypothalamic neurogenesis might influence the peripheral components of physiological processes, such as systemic aging, through the transport of the products of neurosecretion to the periphery through the CVOs, as well as through the transfer of neural/neuroendocrine cell-derived EVs through the CVOs and the BBB [66,398,399]. Interestingly, in the mouse adult hypothalamus, NPCs situated in the wall of the floor of the 3^rd^ ventricle are remarkably enriched with EVs loaded with high concentrations of miRNAs. These miRNAs are also present in the CSF, but their concentration declines with aging [399]. We propose that hypothalamic NPC-derived EVs may (a) act in an autocrine and paracrine fashion, regulating hypothalamic neurogenesis/neural circuits, (b) regulate extrahypothalamic neurogenesis/neural circuits through the CSF and (c) be involved in the communication between the hypothalamus and the periphery of the organism through the CVOs and the BBB [66]. It would be worth exploring whether adult hypothalamic NPC-derived EVs (a) can be internalized by hypothalamic neurons, astrocytes, oligodendrocytes and NPCs, and what are the effects of these EVs on the hypothalamic neurogenic niche and neural circuits, (b) can reach extrahypothalamic neurogenic niches/neural circuits, and what are their effects on them, and (c) can reach the periphery of the organism, and what are their effects on peripheral targets. As there are at least four subpopulations of NPC-like cells in the postmortem adult hypothalamus, it would be worth exploring the differential biosynthesis, content, release, trajectory, targets and functions of EVs derived from these NPCs. As the living human brain is inaccessible to experimentation, we propose the study of hypothalamic NPC-derived EVs in in vitro 3D models of the whole brain and/or hypothalamus [66,157], as well as in human-derived biofluids. We speculate that, in this way, we could gain insights into the role of hypothalamic NPCs in homeostatic regulation (health) and dysregulation (disease). 

## 9. Outstanding Questions

- What is the secretome of the distinct NPC subpopulations in the adult human hypothalamus? What neuropeptides/receptors do these NPCs express; what is their role?
- What is the role of the PACAP/PAC1 system in hypothalamic neurogenesis?
- What are the content and targets of EVs synthesized and released from NPC subpopulations in the adult human hypothalamus? In the opposite direction, what are the origin and effects of EVs that target hypothalamic NPCs?
- What is the role of ependymal cells, ribbon cells, and buds?
- What is the significance of the morphological similarity of suprachiasmatic cells to ribbon cells? What is the role of suprachiasmatic cells?
- Does neurogenesis occur in the adult human PVN? What are the characteristics and role of this neurogenic subniche?
- What is the role of the OVLT in the hypothalamic neurogenic niche?
- What is the role of glial cells (astrocytes, oligodendrocytes and microglia) in the hypothalamic neurogenic niche?
- What is the molecular mechanism through which HFD induces the activation of the NFκΒ pathway in mouse MBH NPCs?
- What is the molecular mechanism through which activation of the NFκΒ pathway in the mouse hypothalamus induces systemic aging?
- Is the secretome of MBH NPCs responsible for systemic aging in humans as in mice?
- What is the role of biological rhythms and their disruption in adult hypothalamic neurogenesis? What is the role of stress?
- Could brain/hypothalamic organoids/assembloids be utilized to study the human hypothalamic neurogenic niche?

## 10. Conclusions

There is evidence that neurogenesis takes place in the postnatal/adult hypothalamus. Although the hypothalamus is highly conserved across species, there are fundamental differences in the organization of the hypothalamic neurogenic niche between rodents and humans. In rodents, NPCs are mainly distinct subpopulations of tanycytes lining the wall of the 3^rd^ ventricle at different levels of the MBH, a region involved in the regulation of core, low-order processes, such as feeding and metabolism. In the human hypothalamus, the neurogenic niche consists of at least four distinct NPC cell-like subpopulations: tanycytes, ependymal, ribbon, and suprachiasmatic cells. Ependymal and ribbon cells are organized in a gap-and-ribbon conformation similar to that in the SVZ. Through ependymal and ribbon cells, the hypothalamic neurogenic niche may be a key regulator of extrahypothalamic neurogenesis and neural circuits. We propose that neurogenesis also takes place in the PVN. In addition, the constitutional presence of intraPVN interneuronal regulatory circuits indicates that the PVN subniche might be the structural equivalent of the SGZ. We propose that ependymal/ribbon cells and the PVN subniche might be involved in the regulation of high-order physiological processes/behaviors specific to humans. As PACAP and PAC1 are highly expressed in the PVN, and PACAP is a known potent regulator of neurogenesis in the SVZ and SGZ, we propose that this neuropeptide may play a central role in hypothalamic neurogenesis as well as in the regulation of extrahypothalamic neurogenesis and neural circuits by the hypothalamus. There are numerous unanswered questions regarding the structure, extent, secretome, functional and evolutionary role of the hypothalamic neurogenic niche. We hypothesize that the utilization of brain/hypothalamic organoids/assembloids—to model adult hypothalamic neurogenesis in humans—and the study of the patterns of intercellular communication through EVs may shed light on the structure and role of the hypothalamic neurogenic niche as well as on the cellular and molecular mechanisms underpinning hypothalamic neurogenesis.

## Abbreviations

ACTH: adrenocorticotropic hormone; AgRP: agouti-related peptide; α-MSH: α-melanocyte-stimulating hormone; AP; area postrema; ARC: arcuate nucleus; ASCL1: Achaete-Scute Family BHLH Transcription Factor 1; AVP: arginine vasopressin; AVPR: arginine vasopressin receptor; AV V-SVZ: anterior ventral ventricular-subventricular zone; BBB: blood-brain barrier; BDNF: brain- derived neurotrophic factor; BLBP: brain lipid-binding protein; BLBP: brain lipid binding protein; BMAL1: Basic Helix-Loop-Helix ARNT Like 1; BMI1: Polycomb complex protein BMI-1; BNST; bed nucleus of the stria terminalis; BrdU: Bromodeoxyuridine; CART: cocaine- and amphetamine-regulated transcript; CB1R: cannabinoid receptor type 1; CCND1: cyclin D1; CeA: central nucleus of the amygdala; CGS: central gray of the spinal cord; CNS: central nervous system; CP: cortical plate; CRE: cAMP response element; CRH: corticotropin-releasing hormone; CRHR: CRH receptor; CRR: counter-regulatory neuroendocrine response; CSF: cerebrospinal fluid; CVO: circumventricular organ; DAT1: dopamine transporter; DCX: doublecortin; DG: dentate gyrus; DIO1: Iodothyronine Deiodinase 1; DIO2: Iodothyronine Deiodinase; DKK1: dickkopf Wnt signaling pathway inhibitor 1; DLK1: Delta Like Non-Canonical Notch Ligand 1; DLX2: distal-less homeobox 2; DMN: dorsomedial nucleus; DMV: dorsal motor nucleus of the vagus; DVC: dorsal vagal complex; EGFP: enhanced green fluorescent protein; ERα: estrogen receptor alpha; ESCs; embryonic stem cells; EV: extracellular vesicle; FEZF2: EZ family zinc finger 2; FGF: fibroblast growth factor; FSH: follicle-stimulating hormone; GAD: glutamic acid decarboxylase; GC: glucocorticoid; GDGF: glial cell line-derived growth factor; GFAP: glial fibrillary acidic protein; GH: growth hormone; GHRH: growth hormone-releasing hormone; GLAST: glutamate aspartate transporter; GLP1: glucagon-like peptide-1; GLUT2: glucose transporter 2; GnRH: gonadotropin-releasing hormone; GRP: gastrin-releasing peptide; HCRT: hypocretin; HES5: Hes Family BHLH Transcription Factor 5; HFD: high-fat diet; HMGB1: high-mobility group box 1; HP1: heterochromatin protein 1; HPS: hypophyseal portal system; HPT: hypothalamic-pituitary-thyroid; HUVEC: human umbilical endothelial cell; IFNγ: interferon γ; IGF1: insulin growth factor 1; IKBKB: Inhibitor of Nuclear Factor Kappa B Kinase Subunit Beta; IKBKE: Inhibitor of Nuclear Factor Kappa B Kinase Subunit Epsilon; IL: interleukin; IML: intermediolateral column of the spinal cord; IKKβ: ΙκΒ kinase; iPSC: induced pluripotent stem cell; IPC: intermediate progenitor cell; ipRGC: intrinsically photosensitive retinal ganglion cell; IRX3: iroquois-class homeodomain protein 3; K_ATP_: ATP-sensitive potassium channel; KP: kisspeptin; LC: locus caeruleus; LepR: leptin receptor; LH: luteinizing hormone: LHA: lateral hypothalamic area; LHX2: LIM Homeobox 2; LPBN: lateral parabrachial nucleus; LPS: lipopolysaccharide; LV: lateral ventricle; MBH: mediobasal hypothalamus; MCH: melanin-concentrating hormone; MCT: monocarboxylate transporter; MC4R: melanocortin receptor 4; ME: median eminence; MFGE8: milk fat globule-EGF factor 8 protein); miRNA: microRNA; MPO: medial preoptic nucleus; MSC: mesenchymal stem cell; MSI1: Musashi RNA binding protein 1; ncRNA: non-coding RNA; ND3: NADH dehydrogenase 3; NE: norepinephrine; NFI: Nuclear factor I; NG2: neuron-glial antigen 2; NKD: neurokinin; NOS1: nitric oxide synthase 1; NPC: Neural progenitor cell; NPY: neuropeptide Y; NPYR: neuropeptide Y receptor; NR2E1: Nuclear receptor subfamily 2 group E member 1; NRF2: NF-E2-related factor-2; NSC: neural stem cell; NFκΒ: Nuclear factor κappa Β; NOTCH1: Neurogenic locus notch homolog protein 1; NTS: nucleus tractus solitarius; OATP1C1: Organic Anion Transporter Family Member 1C1; OB: olfactory bulb; OC: optic chiasm; OCT4: octamer-binding transcription factor 4; OLIG2: oligodendrocyte transcription factor; OR: optic recess; Oxt: oxytocin; OXTR: oxytocin receptor; OVLT: organum vasculosum of the lamina terminalis; PACAP: pituitary adenylate cyclase-activating polypeptide; PAC1: PACAP receptor 1; PAG: periaqueductal gray; PAX6: paired box 6; PBN: parabrachial nucleus; PCNA: proliferating cell nuclear antigen; PCSK1N: proprotein convertase subtilisin/kexin type 1 inhibitor; PD: Parkinson’s disease; PDGF: platelet-derived growth factor; PER: period; PH: posterior hypothalamus; PNS: peripheral nervous system; POMC: proopiomelanocortin; PPII: pyroglutamyl peptidase II; PROX1: prospero homeobox 1; PRSS56: Serine Protease 56; PSD95: postsynaptic density protein 95; PT: pars tuberalis; PVN: paraventricular nucleus of the hypothalamus; RAX: Retina And Anterior Neural Fold Homeobox Protein; RBFOX3: the RNA binding Fox-1 homolog 3; REM: rapid eye movement; RGC: radial glial cell; RHT: retinohypothalamic tract; RLGC: radial-like glial cell; RMS: rostral migratory stream; ROS: reactive oxygen species; RVLM: rostroventrolateral medulla; RVM: rostroventral medulla; SCN: suprachiasmatic nucleus; SCO: subcommissural organ; SDNSF: stem cell-derived neural stem/progenitor cell supporting factor; SF1: steroidogenic factor-1; SFO: subfornical organ; SGK1: serum- and glucocorticoid-inducible kinase 1; SGZ: subgranular zone; Shh: Sonic hedgehog; SIX6: SIX Homeobox 6; SOCS3: Suppressor of Cytokine Signaling 3; SON: supraoptic nucleus; SOX2: SRY-box transcription factor 2; SP: subplate; SPRR1A: Small Proline Rich Protein 1A; SPZ: subparaventricular zone of the hypothalamus; STAT1: signal transducer and activator of transcription 1; SVZ: subventricular zone; SuM: supramammillary nucleus; TASR1: Taste Receptor Type 1 Member 2; TBI: traumatic brain injury; TBR2: T-box brain protein 2; TBX3: T-Box Transcription Factor 3; TH2: tyrosine hydroxylase 2; TLR: Toll-like receptor; TLX: Tailless like receptor; TNFα: tumor necrosis factor alpha; TRH: thyrotropin-releasing hormone; TrkB; tropomyosin receptor kinase B; TSH: thyroid-stimulating hormone; TTFL: transcriptional-translational feedback loop; VEGF: vascular endothelial growth factor; VGLUT: vesicular glucose transporter; VIP: vasoactive intestinal peptide; vlPAG: ventrolateral periaqueductal gray; VLPO: ventrolateral preoptic nucleus; VMAT2: vesicular monoamine transporter 2; VMN: ventromedial nucleus; VPAC1: vasoactive intestinal peptide receptor 1; VPAC2: vasoactive intestinal peptide receptor 2; VZ: ventricular zone.
